# Mental health and its consequences in people living with HIV: A network approach

**DOI:** 10.1002/brb3.70021

**Published:** 2024-10-20

**Authors:** Elise M. G. Meeder, Louise E. van Eekeren, Marc J. T. Blaauw, Albert L. Groenendijk, Wilhelm A. J. W. Vos, Jan van Lunzen, Leo A. B. Joosten, Mihai G. Netea, Quirijn de Mast, Willem L. Blok, Annelies Verbon, Marvin A. H. Berrevoets, Vasiliki Matzaraki, Andre J. A. M. van der Ven, Arnt F. A. Schellekens

**Affiliations:** ^1^ Department of Psychiatry, Radboudumc Radboud University Nijmegen The Netherlands; ^2^ Donders Institute for Brain Cognition and Behavior Radboud University Nijmegen The Netherlands; ^3^ Nijmegen Institute for Scientist‐Practitioners in Addiction (NISPA) Radboud University Nijmegen The Netherlands; ^4^ Department of Internal Medicine and Infectious Diseases, Radboudumc Radboud University Nijmegen The Netherlands; ^5^ Department of Internal Medicine and Infectious Diseases Elizabeth‐Tweesteden Ziekenhuis Tilburg The Netherlands; ^6^ Department of Internal Medicine and Department of Medical Microbiology and Infectious diseases, Erasmus Medical Center (MC) Erasmus University Rotterdam The Netherlands; ^7^ Department of Internal Medicine and Infectious Diseases OLVG Amsterdam The Netherlands; ^8^ Department of Medical Genetics Iuliu Hatieganu University of Medicine and Pharmacy Cluj‐Napoca Romania; ^9^ Department of Immunology and Metabolism, Life and Medical Sciences Institute University of Bonn Bonn Germany

**Keywords:** HIV, network analysis, psychiatry, quality of life, substance use

## Abstract

**Objectives:**

Psychiatric symptoms occur frequently in people living with human immunodeficiency virus (PLWH), which may affect quality of life, sexual risk behavior, and adherence to antiretroviral therapy (ART). Data from large cohorts are limited, and symptoms are often analyzed in isolation. Therefore, we applied a network analysis to assess the interrelatedness of mental health indicators in a large cohort of PLWH.

**Methods:**

We included 1615 PLWH on ART. Participants reported on the severity of depression, anxiety, impulsivity, substance use, quality of life, sexual risk behavior, and ART adherence. An Ising network model was constructed to analyze interrelations between mental health indicators and connections with clinical consequences.

**Results:**

Our network analysis revealed that symptoms of depression, anxiety, and indicators of impulsivity were interrelated. Substance use was prevalent and strongly connected with sexual risk behavior. Quality of life was most strongly connected with symptoms of depression. Unexpectedly, ART adherence did not display connections with any of the mental health indicators.

**Conclusion:**

In PLWH, the interrelatedness between symptoms of depression and anxiety and indicators of impulsivity is high. Mainly, depressive symptoms seem to impact quality of life, which warrants attention for depression in PLWH. We did not observe evidence for the common assumption that patients suffering from psychiatric symptoms are less adherent to HIV treatment.

## Graphical abstract

Psychiatric symptoms occur frequently in people living with HIV (PLWH). This study shows that in PLWH, depression, anxiety, and impulsivity are interrelated. Importantly, quality of life in PLWH is strongly connected to depression. However, we found no relation between any mental health indicator and the adherence to HIV treatment.

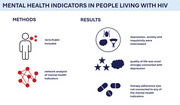

## 1 INTRODUCTION

People living with HIV (PLWH) report higher levels of mental health issues as compared to the general population (Chander et al., 2006; Himelhoch et al., 2009). For example, with a prevalence of around 10% for depression (Ciesla & Roberts, 2001) and up to 40% for alcohol use disorder (Samet et al., 2004), these conditions occur twice as frequently in PLWH as in the general population (Ciesla & Roberts, 2001; Samet et al., 2004). The increased prevalence of psychiatric diseases in PLWH has several potential consequences. First of all, the presence of a psychiatric disorder, depression in particular, has been shown to have a major impact on quality of life in PLWH (Asrat et al., 2020). Second, psychiatric symptoms, such as depression and substance use, have been associated with suboptimal adherence to antiretroviral therapy (ART) in PLWH (Asrat et al., 2020; Mayston et al., 2012). Furthermore, psychiatric symptoms, specifically depression, impulsivity, and substance use, have been associated with sexual risk behavior in PLWH (Arends et al., 2018; Meade & Sikkema, 2005). However, the relationship between psychiatric symptoms and the aforementioned consequences has not yet been fully disentangled.

In order to study psychiatric symptomatology in PLWH, it is important to take into account that psychiatric disorders in general are highly heterogeneous. Patients suffering from the same disorder often exhibit a relatively unique pattern of symptomatology (Fried & Nesse, 2015). Furthermore, it has been shown that individual psychiatric symptoms are differentially related to predisposing risk factors and clinical outcomes, such as impaired functioning (Fried et al., 2014, 2016). Therefore, when studying the potential consequences of psychiatric symptoms in PLWH, focusing on individual symptoms instead of diagnostic classifications or questionnaire sum scores can provide important insights. Furthermore, various psychiatric symptoms frequently co‐occur in PLWH, as is the case in the general population (Burnam et al., 2001; Kessler, 1994; Van de Wijer et al., 2021). For instance, in PLWH, substance use is known to frequently coincide with symptoms of depression and anxiety (Burnam et al., 2001; Van de Wijer et al., 2021). A possible explanation for the frequent co‐occurrence of diverse psychiatric symptoms is that psychiatric symptoms themselves influence one another (Borsboom & Cramer, 2013; Epskamp et al., 2018; Fried et al., 2017). For example, worrying might cause sudden feelings of panic, panic attacks might drive excessive use of alcohol, and excessive alcohol use might cause anhedonia.

Network analysis is a promising approach that takes into account interrelationships among multiple psychiatric symptoms (Borsboom & Cramer, 2013; Epskamp et al., 2018; Fried et al., 2017). According to the network theory, central symptoms are more likely to induce other symptoms and might therefore play a key role in the onset and maintenance of a disorder (Van Borkulo et al., 2014). The use of network analysis in patients with depression has, for instance, shown that the most central depression symptoms (i.e., sadness, anhedonia, energy loss, and concentration problems) also had the biggest impact on impaired functioning (Fried et al., 2014, 2016). Therefore, targeting central symptoms may result in interventions with better clinical outcomes (Fried et al., 2016), also in PLWH populations.

Application of network analysis in PLWH can explain the co‐occurrence of various psychiatric symptoms in PLWH by showing how different symptoms are connected. Furthermore, it may identify treatment targets specific for this population, by revealing central psychiatric symptoms and symptoms with the biggest impact on quality of life. Finally, it could point at possibilities for the improvement of HIV prognosis and prevention of HIV transmission, by demonstrating which psychiatric symptoms are linked to reduced ART adherence and sexual risk behavior. However, up to date, very few network analyses on psychiatric symptoms in PLWH have been performed (Han et al., 2023; Wen et al., 2023; Zhu et al., 2022). Importantly, these studies had several limitations. One was in a relatively small sample (Han et al., 2023), and the two other studies had overlapping samples (Wen et al., 2023; Zhu et al., 2022). Furthermore, all of these studies were in the Chinese context and did not include potential consequences of mental health issues in HIV, such as impact on quality of life and therapy adherence. Yet, their findings do demonstrate the relevance of a network approach to mental health in PLWH. Therefore, this approach needs to be expanded to non‐Chinese populations.

The current study aimed to investigate mental health networks in PLWH on ART. First, we describe the prevalence of symptoms of depression, anxiety, and indicators of impulsivity and substance use in 1615 PLWH on ART. Next, we used a network analysis to (1) analyze interrelationships between these symptoms and indicators, (2) identify which of these symptoms and indicators are most central, and (3) explore associations between the mental health indicators and clinical consequences, including quality of life, ART adherence, and sexual risk behavior.

## 2 METHODS

### 2.1 Design

The 2000HIV study consists of a prospective observational cohort of PLWH on long‐term ART (Vos et al., 2022). In the present study, only baseline data were used. All participants provided written informed consent prior to participation. The study protocol was approved by the independent regional ethical review board Arnhem Nijmegen (ref. NL68056.091.81), published at clinicaltrials.gov (ID: NCT03994835). The study was conducted in accordance with the principles of the Declaration of Helsinki.

### 2.2 Participants

PLWH were recruited from October 2019 till October 2021 from four Dutch HIV treatment centers (Radboudumc Nijmegen, Erasmus MC Rotterdam, OLVG Amsterdam, Elisabeth‐Tweesteden Ziekenhuis Tilburg), as has been described before (Vos et al., 2022). Inclusion criteria were as follows: proven HIV‐1 infection, age ≥18 years, duration of ART ≥ 6 months, latest HIV‐1 RNA ≤200 copies/mL. Exclusion criteria were current pregnancy, viral hepatitis B or C, the presence of acute infection, and severe communication problems (e.g., due to language barriers). In the present study, patients with spontaneous HIV control without ART were not included, because ART therapy adherence could not be measured in this subgroup.

### 2.3 Instruments

Sociodemographic and clinical data were measured by using structured questionnaires and extracting information from electronic patient records and collected in Castor EDC.

Symptoms of depression and anxiety were measured using the Hospital Anxiety and Depression Scale (HADS) (Zigmond & Snalth, 1983). The HADS is a 14‐item self‐report questionnaire, developed to detect states of anxiety and depression in the medical setting (Zigmond & Snalth, 1983). The HADS consists of an anxiety and a depression scale, with scores ranging from 0 to 21. Physical symptoms of anxiety or depression, which are likely to be caused by physical illness (e.g., weight loss), are not included in the HADS (Zigmond & Snalth, 1983). The HADS has been used in the context of a variety of somatic diseases, including HIV (Channer et al., 1985; Lewis, 1991; Moorey et al., 1991). Its psychometric properties (Spinhoven et al., 1997) and its dimensional structure and reliability have been proven to be stable across different medical settings and age groups (Spinhoven et al., 1997).

Indicators of impulsivity were assessed through the Barratt Impulsiveness Scale (BIS‐11) (Barratt, 1985). The BIS‐11 consists of 30 statements, such as “I say things without thinking” (indicative of high impulsivity) or “I am future‐oriented” (indicative of low impulsivity). Patients rated the frequency in which each statement applied to them, using a 4‐point Likert scale ranging from 1 (“rarely/never”) to 4 (“almost always”). Three higher order BIS‐11 factors have previously been identified: attentional impulsivity (the inability to focus and the tendency to make rapid decisions), motor impulsivity (the tendency to act quickly), and nonplanning impulsivity (the inability to plan for the future) (Patton et al., 1995). The BIS‐11 has been extensively used in the field of impulsivity research (Chang et al., 2017; Kjome et al., 2010), including in cohorts of PLWH (Arends et al., 2018; Meeder et al., 2021), and has a good internal consistency and retest reliability (Patton et al., 1995; Stanford et al., 2009). The internal consistency of all self‐questionnaires within our sample can be found in Table S1.

Substance use was assessed using the Measurements in the Addictions for Triage and Evaluation (MATE)‐Q parts 1a and 1b (Oudejans et al., 2020). For the network analysis, only part 1b of the MATE‐Q was used. This part consists of 10 items, each exploring whether and how often a patient used a specific drug of abuse during the past 30 days: alcohol, tobacco, cannabis, opioids, cocaine, ecstasy, other stimulants (e.g., amphetamines, khat, and speed), sedatives, gambling, and a rest category of other drugs (e.g., psilocybin, GHB, and poppers). Of these 10 items, the following were not included in the final network analysis, due to negligible prevalence rates: opioids, sedatives, gambling, and “other stimulants” (e.g., amphetamines, khat, and speed). For alcohol use, heavy use was defined as >21 standard glasses weekly in men and >14 standard glasses weekly in women. The MATE‐Q has been demonstrated to have sufficient concurrent validity with the MATE 2.1 structured clinical interview (Oudejans et al., 2020), which is being used in multiple European countries and has adequate psychometric properties (Buchholz et al., 2011; Galland et al., 2018; Hell et al., 2018).

Quality of life was measured by the EuroQol 5‐dimension 5‐level questionnaire (EQ‐5D‐5L) (Herdman et al., 2011). The EQ‐5D‐5L is the third and newest version of the EQ‐5D. Like its previous version, it consists of five dimensions: mobility, self‐care, usual activities, pain/discomfort, and anxiety/depression (EuroQol Research Foundation, 2019). In the EQ‐5D‐5L, each of these dimensions consists of five response levels: no problems, slight problems, moderate problems, severe problems, and extreme problems. Due to the increased number of response levels as compared to the EQ‐5D‐3L, the EQ‐5D‐5L has increased reliability and sensitivity (EuroQol Research Foundation, 2019; Herdman et al., 2011). The EQ‐5D‐5L is often used, with good psychometric properties, also in HIV populations (Cooper et al., 2017; Tran et al., 2012).

ART adherence was measured by the Morisky Medication Adherence Scale‐8 (MMAS‐8) (Morisky et al., 2008). The MMAS‐8 consists of seven yes/no questions (rated 1/0) and one question with a five‐point Likert‐scale (rated 0–4). A total score of ≥6 is indicative of therapy nonadherence. The MMAS‐8 is an updated version of the original MMAS (Morisky et al., 1986), to which four items were added addressing the circumstances surrounding adherence behavior. The MMAS‐8 has been used extensively to assess adherence in clinical trials in various chronic diseases (Arora et al., 2014; Berry et al., 2015; Bramlage et al., 2014; Park et al., 2014). It has also been used in PLWH (Biney et al., 2021; Willoughby et al., 2021) and has adequate internal consistency and reproducibility (Moon et al., 2017).

Sexual risk behavior was measured by questions on the number of sexual partners during the past year and the number of sexually transmitted diseases (STDs) during the past year, which were part of a larger self‐questionnaire on sociodemographic and lifestyle features.

### 2.4 Statistical analysis

Descriptive statistics were used for describing population characteristics. For continuous variables, we calculated means and standard deviations, and for categorical variables, we calculated numbers and percentages. Variables included in the network analysis with missing values were imputed using median imputation. The rate of missingness was low, with a maximum missing percentage of 3.9% for item B16 (“I change jobs”).

For the network analysis, we performed an Ising model analysis to assess the interactions between psychiatric symptoms and indicators of impulsivity and substance use, using the R package *IsingFit* (v0.3.1; Van Borkulo et al., 2014). Likert‐scale items on mental health questionnaires are ordinal variables, which often display a highly skewed distribution (Epskamp, 2017). In the field of network modeling, no consensus regarding the optimal method of handling such data has yet been reached (Epskamp, 2017; Van Borkulo et al., 2014). Following previous studies (Cai et al., 2022; Van Borkulo et al., 2014), we, therefore, opted to dichotomize the values of all BIS‐11, HADS, MATE‐Q, and EQ‐5D‐5L items, indicating whether or not a certain symptom/problem was present or absent. For BIS‐11 items, Rates 1 (“rarely/never”) and 2 (“sometimes”) were converted to 0 (“not present”), as sometimes does not justify the indicator impulsivity to be present. Rates 3 (“frequently”) and 4 (“almost always”) were converted to 1 (“present”). For HADS items, Rate 0 remained 0 (“symptom not present”) and Rates 1, 2, and 3 were converted to 1 (“symptom present”). For the MMAS‐8, all items except 1 were already dichotomous. For the only MASS‐8 item with a Likert scale (T8: “I have difficulty remembering to take all my prescribed medication”), Rate 0 (“never”) remained 0 (“symptom not present”), and Rates 1 (“rarely”), 2 (“once a month”), 3 (“once a week”), and 4 (“daily”) were converted to 1 (“symptom present”). For the EQ‐5D‐5L items, the response level referring to “no problems” remained 0 (“symptom not present”). Response levels “slight problems,” “moderate problems,” “severe problems,” and “extreme problems” were all converted to 1 (“symptom present”). A detailed overview of how the dichotomization affected the frequencies of variable levels is presented in Table S2.

An Ising model is an undirected network, consisting of nodes representing binary variables, and edges representing conditional dependencies between the nodes. These dependencies are calculated using logistic regression. If variables A and B are connected by an edge, they are related even after controlling for all other variables in the network. In other words, the relationship between A and B cannot be explained by any of the other variables in the network. Conversely, if two variables are not connected, they are not directly related. The importance of individual nodes in a network can be assessed by measuring node centrality, for which multiple centrality measures exist. The centrality index s*trength* is most suited to measure centrality in mental health networks (Bringmann et al., 2019). Node strength depicts how strongly a node is directly connected to the other nodes, by adding up the strengths of all edges connected with the node.

Due to sampling variation, any network can contain false positive associations, also known as *spurious* connections. To control for spurious connections, the regularization technique *eLASSO* was used. The *eLASSO* (Van Borkulo et al., 2014) procedure, implemented in the *IsingFit* package, combines regularized logistic regression with model selection based on the extended Bayesian information criterion (EBIC). The regularized logistic regression estimates a range of networks, ranging from fully connected to fully disconnected. Next, the best network is selected using EBIC. EBIC favors solutions that assign fewer neighbors to a given node. EBIC contains a parameter γ, which controls the strength of the penalty on the number of neighbors, that is, how much EBIC prefers simpler models. EBICs of both 0.25 and 0.5 are common thresholds in network analyses. In this study, we opted to apply *γ* = 0.25, because we wanted to reduce the risk of omitting true edges from the network (i.e., Type 2 errors). Furthermore, we applied the AND rule to determine the final edge set. The AND rule requires both regression coefficients from the regularized logistic regression of variable A on C and of C on A to be nonzero, which also results in a lower number of estimated connections.

In order to assess the accuracy of the network, the accuracy of the edge weights and the differences in edge weights and node strengths were estimated by calculating bootstrapped confidence intervals, and the stability of the order of node strength was assessed by case‐dropping subset bootstrap (Epskamp et al., 2018). For each of the accuracy analyses, the R package *bootnet* was used and 1000 permutations were performed. R version 4.0.5 (R Core Team) was used for all analyses.

## 3 RESULTS

The demographic, psychiatric, and somatic characteristics of the cohort are presented in Table 1 . Additional somatic characteristics of the entire 2000HIV cohort were described elsewhere (Vos et al., 2022). A minority of the participants was female (13.7%), and the mean age was 52.0 (±11.6) years. The most frequent mode of transmission was through sexual intercourse between men who have sex with men (MSM) (74.8%). The mean duration since HIV diagnosis was 13.8 (±8.2) years, and the mean duration on ART was 11.5 (±6.7) years. The most frequently used substance was tobacco: 28.7% of the participants currently smoked, and the mean number of pack‐years was 25.2 (±22). The percentage of heavy alcohol use was 11.1 for the past month and 16.2 during lifetime. During the past month, 13.5% used cannabis, 5.9% used cocaine, 9.2% used ecstasy, and 8.0% used other drugs that mainly involved GHB and/or poppers. According to the HADS and BIS‐11 scores, 6.1% suffered from severe depression symptoms, 9.3% from severe anxiety symptoms, and 8.4% could be classified as being impulsive.

**TABLE 1:** Characteristics of the patients from the 2000HIV cohort included in this study (*N* = 1615).

General and HIV‐related characteristics		Depression and anxiety (HADS)		Substance use (MATE‐Q)^b^	
Age (years)	52.0 ± 11.6	Total score (0–42)	8.5 ± 7.0	Alcohol, heavy^c^	179 (11.1%)
Female sex	221 (13.7%)	Mild symptoms	133 (8.2%)	Tobacco	463 (28.7%)
HIV duration (years)	13.8 ± 8.2	Severe symptoms	279 (17.3%)	Packyears	25.2 ± 22
Time on cART (years)	11.5 ± 6.8	Anxiety	4.6 ± 4.0	Cannabis	218 (13.5%)
Nadir CD4 (cells/mm^3^)	250 (140–380)	Mild symptoms	204 (12.6%)	Cocaine	95 (5.9%)
Current CD4 (cells/mm^3^)	708 (540–903)	Severe symptoms	150 (9.3%)	Ecstasy	148 (9.2%)
Latest viral load undetect	1133 (91.6%)	Depression	3.9 ± 3.7	Other stimulants	52 (3.2%)
MSM^a^	1208 (74.8%)	Mild symptoms	177 (11.0%)	Sedatives	49 (3.0%)
**Psychiatric medical history**		Severe symptoms	98 (6.1%)	Opioids	10 (0.6%)
Depressive disorder	207 (12.8%)	**Impulsivity (BIS‐11)**		Other drugs	130 (8.0%)
Substance use disorder	128 (7.9%)	Total score (30–120)	59.3 ± 8.7	**Quality of life (EQ‐5D‐5L)** ^c^	
Anxiety disorder	58 (3.6%)	Highly impulsive	135 (8.4%)	VAS (0–100)	78.7 (15.0)
Personality disorder	42 (2.6%)	Motor	20.7 ± 3.5	Problems with …	
**Prescribed psychiatric medication**		Attentional	15.6 ± 3.4	Mobility	305 (18.9%)
Benzodiazepines	85 (5.3%)	Nonplanning	23.0 ± 4.4	Selfcare	43 (2.7%)
Antidepressants	101 (6.3%)			Usual activities	275 (17.0%)
Antipsychotics	27 (1.7%)			Pain/discomfort	707 (43.8%)
Stimulants	11 (0.7%)			**Therapy adherence (MMASS‐8)**	
Other	34 (2.1%)			High	720 (44.6%)
				Medium	595 (36.8%)
				Low	300 (18.6%)
				**Sexual risk** behavior	
				STD in past year	350 (21.7%)

*Note*: For continuous outcomes, mean ± SD and for categorical outcomes, *N* (%) are depicted.

Abbreviations: BIS‐11, Barratt Impulsiveness Scale; BMI, body mass index; cART, combination antiretroviral therapy; EQ‐5D‐5L, EuroQol 5‐dimension 5‐level questionnaire; HADS, Hospital Anxiety and Depression Scale; MATE‐Q, Measurements in the addictions for triage and evaluation Q; MMAS‐8, Morisky medication adherence scale‐8; STD, sexually transmitted disease; VAS, visual analog scale.

^a^ Transmission by sexual intercourse between men who have sex with men.

^b^ Use of substance during the past 30 days.

^c^ Heavy alcohol use was defined as >21 (♂)/14 (♀) alcohol units/week. For the EQ‐5D‐5L, problems with anxiety/depression are not included, as these have been measured more thoroughly using the HADS.

**TABLE 2:** Network node abbreviations.

**HADS**	
**Depression** H2: I still enjoy the things I used to enjoy^a^ H4: I can laugh and see the funny side of things^a^ H6: I feel cheerful^a^ H8: I feel as if I am slowed down H10: I have lost interest in my appearance H12: I look forward with enjoyment to things^a^ H14: I can enjoy a good book or radio or TV^a^	**Anxiety** H1: I feel tense or “wound up” H3: I get a sort of frightened feeling as if something awful is about to happen H5: Worrying thoughts go through my mind H7: I can sit at ease and feel relaxed^a^ H9: I get a sort of frightened feeling like “butterflies” in the stomach H11: I feel restless as I have to be on the move H13: I get sudden feelings of panic
**BIS‐11**	
**Motor impulsivity—motor** B2: I do things without thinking B3: I make up my mind quickly B4: I am happy‐go‐lucky B16: I change jobs B17: I act “on impulse” B19: I act on the spur of the moment B21: I change residences B22: I buy things on impulse B23: I can only think about one thing at a time B25: I spend or charge more than I earn B30: I am future oriented^a^ **Nonplanning impulsivity** B1: I plan tasks carefully^a^ B7: I plan trips well ahead of time^a^ B8: I am self‐controlled^a^ B10: I save regularly^a^ B12: I am a careful thinker^a^	B13: I plan for job security^a^ B14: I say things without thinking B15: I like to think about complex problems^a^ B18: I get easily bored when solving thought problems B27: I am more interested in the present than the future B29: I like puzzles^a^ **Attentional impulsivity** B5: I do not “pay attention” B6: I have “racing” thoughts B9: I concentrate easily B11: I “squirm” at plays or lectures B20: I am a steady thinker B24: I change hobbies B26: I often have extraneous thoughts when thinking B28: I am restless at the theater or lectures
**MATE‐Q**	
AL: Heavy alcohol use during the past month SM: Current smoking CA: Cannabis use during the past month CO: Cocaine use during the past month	XT: Ecstasy use during the past month OT: Use of other drugs during the past month (e.g., GHB, psilocybin, and poppers)
**EQ5D‐5L**	
QV: Visual analog scale on perceived health status QP: Pain and discomfort problems QM: Mobility problems	QS: Self‐care problems QA: Usual activity problems
**MMAS‐8**	
T1: I sometimes forget my HIV medication T2: I did not take my HIV medication any day during the past 30 days T3: I stopped taking my HIV medication without telling my doctor T4: I forget to bring my HIV medication when travelling T5: I took my HIV medication according to schedule in the past week^a^	T6: I stopped taking my HIV medication when I felt my HIV infection was controlled T7: I feel hassled about sticking to my HIV medication schedule T8: I have difficulty remembering to take all my prescribed medication
**Sexual risk** behavior	
PR: Multiple sexual partners during the past year	ST: Sexually transmitted disease during the past year

*Note*: “a” denotes reverse score. For MMAS‐8, the original text of the questionnaire items is in this legend summarized into a short statement. Abbreviations: BIS, Barratt Impulsiveness Scale; HADS, Hospital Anxiety and Depression Scale; MATE, Measurements in the Addictions for Triage and Evaluation; MMAS, Morisky Medication Adherence Scale.

**FIGURE 1:**
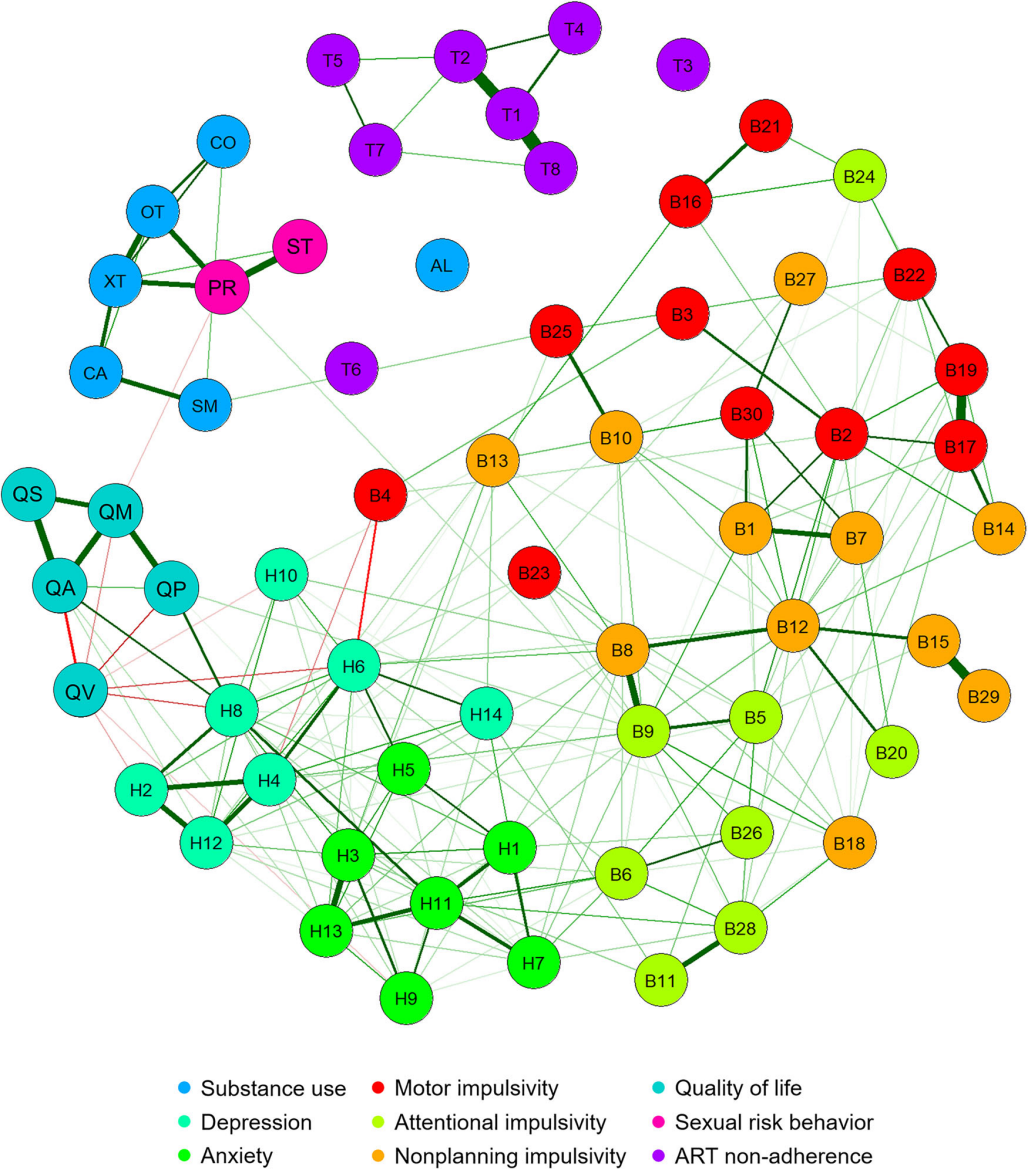
Network of symptoms of depression (Hospital Anxiety and Depression Scale [HADS]), anxiety (HADS), indicators of impulsivity (Barratt Impulsiveness Scale [BIS]‐11), substance use (Measurements in the Addictions for Triage and Evaluation [MATE]‐Q), quality of life (EuroQol 5‐dimension 5‐level questionnaire [EQ‐5D‐5L]), antiretroviral therapy (ART) nonadherence (Morisky Medication Adherence Scale [MMAS]‐8), and sexual risk behavior in the 2000HIV cohort. For the legend of the node abbreviations, see Table 2.

The network of symptoms of depression, anxiety, indicators of impulsivity, and substance use is presented in Figure 1. Visual inspection of Figure 1 reveals that symptoms of anxiety and depression were strongly connected. Symptoms of depression and anxiety were also connected with indicators of impulsivity. Substance use was mainly connected with sexual risk behavior. In detail, ecstasy use and use of other illicit drugs (e.g., psilocybin, gamma hydroxybutyrate https://doi.org/10.1016/j.psychres.2009.11.024and poppers) during the past month were connected with a higher number of sexual partners (edge weights 1.09 and 1.14, respectively, see Table S3), which in turn was connected with the presence of STDs during the past year (edge weight 1.44, Table S3).

Quality of life displayed a relatively high number of connections with symptoms of depression: four out of seven symptoms of depression were connected with quality of life indicators. Quality of life also displayed several connections with symptoms of anxiety. Depressive symptom H8 “the feeling of being slowed down” (i.e., subjective retardation) was the key symptom that connected quality of life (edge weight −0.35, Table S3) with symptoms of depression and anxiety. With a strength centrality index of 5.72, the feeling of being slowed down was also among the most central symptoms within the network (see Figure S1 and Table S4).

The top five nodes in terms of high strength were impulsivity indicator B9 (“I concentrate easily”), depressive symptom H11 (“I feel restless as I have to be on the move”), ART nonadherence indicator T1 (“I sometimes forget my HIV medication”), depressive symptom H8 (“I feel as if I am slowed down”), and impulsivity indicator B12 (“I am a careful thinker”).

Notably, ART adherence was not connected with any of the other nodes in the network. All edge weights can be found in Table S3.

The strength centrality index of the network had an excellent core stability coefficient of 0.75, meaning that 75% of the cases could be dropped without significant changes in the network structure (Figure S3). Furthermore, bootstrapped 95% confidence intervals of the estimated edge weights showed that the estimates were relatively stable (Figure S2). Moreover, bootstrapped difference tests showed that many of the differences between edge weights and node strengths were statistically significant (*p* < .05, see Figures S4 and S5).

## 4 DISCUSSION

In this study, we described the prevalence of psychiatric symptoms and substance use in PLWH and studied interactions between these symptoms and possible clinical consequences using network analysis. In our cohort, we found a high occurrence of substance use and considerable levels of depression, anxiety, and impulsivity. The network demonstrated that of all behavioral symptoms and indicators measured, symptoms of depression and anxiety were most strongly interconnected. Quality of life was mainly connected to symptoms of depression, and sexual risk behavior was connected to substance use. Notably, therapy adherence did not display any connections with depression, anxiety, impulsivity, or substance use.

In‐line with other studies, the levels of substance use in our cohort are substantially higher as compared to the general Dutch population, as is shown by the past month prevalence rates of 29.5% versus 20% for tobacco use, 11.1% versus 7.3% for heavy alcohol use, 13.6% versus 7.3% for cannabis use, 9.2% versus 1% for ecstasy use, and 5.9% versus 0.8% for cocaine use (Trimbos Institute, 2022). For example, in a large Danish study, the proportion of current smokers was more than twice as high among PLWH, as compared to the general population (Helleberg et al., 2013). Similarly, multiple previous studies found a higher prevalence of heavy alcohol use in PLWH (Galvan et al., 2002; Sebit et al., 2004; Shaffer et al., 2004). Given the cross‐sectional nature of our data, we cannot infer any temporal or causal relationship between substance use and HIV infection.

Levels of depression and anxiety in our cohort are slightly higher as compared to the general Dutch population (Spinhoven et al., 1997) and similar to cohorts of PLWH in non‐western countries (Reda, 2011; Wouters et al., 2012). A recent meta‐analysis demonstrated that the prevalence of depressive disorders in PLWH is nearly two times higher than in HIV‐negative subjects (Ciesla & Roberts, 2001). Levels of impulsivity in our PLWH cohort were similar to those in general population samples (Reise et al., 2013; Stanford et al., 2009). These observations may be explained by selection bias, because PLWH with the most severe symptoms of depression or impulsivity might have been less likely to participate in our study.

The network analysis demonstrated that in our cohort, symptoms of depression and anxiety often co‐occur. These findings are in‐line with previous observations of a high level of depression and anxiety comorbidity both in PLWH (Gaynes et al., 2008) and in the general population, where 60% of patients with a current depression also suffer from a current anxiety disorder and vice versa (Lamers et al., 2011). We also found a high connectedness of symptoms of impulsivity with symptoms of depression and anxiety. This finding might seem counter‐intuitive, as psychiatric disorders are traditionally classified into internalizing (e.g., depressive and anxiety disorders) versus externalizing disorders (e.g., ADHD and substance use disorders [SUDs]) (Zisner & Beauchaine, 2016). However, the comorbidity of internalizing and externalizing disorders is often seen in the general population (Biederman et al., 2008; Gilliom & Shaw, 2004; Zisner & Beauchaine, 2016). This might partly be explained by common genetic pathways, because internalizing and externalizing disorders share genetic risk factors (Powell et al., 2021).

The network analysis showed that in our cohort of PLWH, quality of life is mainly connected with symptoms of depression. To a lesser extent, quality of life is also associated with anxiety, but not with impulsivity or substance use. Previous network analyses in PLWH also found a central position for depressive symptoms such as sadness and worry (Han et al., 2023; Wen et al., 2023; Zhu et al., 2022). Moreover, our study is in‐line with a large amount of evidence demonstrating that depression is inversely associated with quality of life in PLWH across the world, in both men and women, and across different age groups (Araújo et al., 2021; Jain et al., 2021; Luseno et al., 2021; Xiaowen et al., 2018). Our study highlights once more that in order to improve quality of life in PLWH, we should focus on the prevention, detection, and treatment of symptoms of depression (Okimat et al., 2022).

In our cohort, the use of ecstasy and other drugs (among which were poppers and GHB) is connected with STDs and with a higher number of sexual partners. The relation between the use of these potentially sex‐enhancing drugs and sexual risk behavior is also observed in the general population, where higher levels of ecstasy use were associated with condomless sex with non‐steady partners, and higher levels of syphilis and hepatitis C (Hampel et al., 2020). Whether substance use could serve as a target for the prevention of STDs requires further research. Future studies on this topic should assess sexual risk behavior more extensively and, for instance, also include condom use and the use of substances during sex (“chemsex”).

Our network analysis showed barely any connections between indicators of impulsivity and substance use. This is in contrast with a large body of evidence showing high levels of impulsivity in patients with various SUDs and an association between impulsivity and substance use (Coffey et al., 2003; Fishbein et al., 2007; Mitchell et al., 2005; Verdejo‐García et al., 2007). However, a previous network analysis among treatment‐seeking patients with SUD also did not find a relationship between impulsivity and substance use (López‐Toro et al., 2022). It has been hypothesized that impulsivity mainly plays a role in the initiation of substance use, and less in later phases of regular substance use (Kreek et al., 2005). Given the mean age (52 years) of the participants in our cohort, it is unlikely that they recently initiated substance use.

It is noteworthy that ART adherence did not display a connection with any psychiatric symptom or the use of any substance. Conversely, multiple previous studies have found suboptimal ART adherence among PLWH who use substances (Gonzalez et al., 2011; Lucas et al., 2001, 2002). However, other studies demonstrated that PLWH who use substances have the same level of adherence as compared to non‐substance using PLWH (Crystal et al., 2001; Malta et al., 2008). Interestingly, in a recent study among Dutch patients with viral hepatitis, a history of SUD was even associated with better hepatitis healthcare utilization (Von den Hoff et al., 2022). These findings falsify the general assumption that people using substances are less therapy adherent. Therefore, healthcare policies should be aimed at reducing stigma and securement of equal access to somatic healthcare for all PLWH, including those who use substances.

This study has several strengths and limitations. The network approach has allowed us to perform an in‐depth, hypothesis‐free assessment of the interrelatedness of psychiatric symptoms, other behavioral indicators, and their clinical consequences in PLWH. Furthermore, the 2000HIV cohort is a highly representative large cohort of PLWH on ART living in a western country. Due to the cross‐sectional design of the study, we were not able to perform a directed network analysis, and therefore, no conclusions regarding the causality of connections can be drawn. Finally, although participants were assessed using an extensive number of validated questionnaires, sexual risk behavior was only assessed by two items. For more insight into the relation between sexual risk behavior, impulsivity, and substance use, network analyses, including scales such as the Sexual Risk Behaviors Scale (Fino et al., 2021), are needed.

In conclusion, this network analysis provides new insights in the interplay of psychiatric symptoms and behavioral indicators in PLWH and has important clinical implications. First, we found that substance use was connected with sexually transmitted infections in PLWH, more than with depression, anxiety, or impulsivity. Substance use may therefore be a relevant target for the prevention of STD transmission. Second, there was no association between any of the psychiatric symptoms and ART nonadherence. Finally, symptoms of depression were the key symptoms influencing quality of life in PLWH. Therefore, targeting symptoms of depression may improve treatment outcomes in PLWH.

## AUTHOR CONTRIBUTIONS

**Elise M. G. Meeder**: Investigation; formal analysis; writing—original draft; visualization; validation; methodology; conceptualization; data curation; software. **Louise E. van Eekeren**: Investigation; writing—review and editing; data curation. **Marc J. T. Blaauw**: Investigation; writing—review and editing; data curation. **Albert L. Groenendijk** and **Wilhelm A. J. W. Vos**: Investigation; writing—review and editing; data curation. **Jan van Lunzen**: Writing—review and editing. **Leo A. B. Joosten** and **Mihai G. Netea**: Funding acquisition; project administration; resources; writing—review and editing. **Quirijn de Mast** and **Willem L. Blok**: Resources; project administration; writing—review and editing. **Annelies Verbon**: Writing—review and editing; project administration; resources. **Marvin A. H. Berrevoets**: Writing—review and editing; project administration; resources. **Vasiliki Matzaraki**: writing—review and editing; methodology; software; formal analysis; supervision. **Andre J. A. M. van der Ven**: Funding acquisition; conceptualization; writing—review and editing; methodology; project administration; supervision; resources. **Arnt F. A. Schellekens**: Supervision; writing—review and editing; methodology; conceptualization.

## CONFLICT OF INTEREST STATEMENT

ViiV Healthcare funded this study but was not involved in data quality control, statistical analyses, or data interpretation. J.L. was previously employed by ViiV Healthcare.

### PEER REVIEW

The peer review history for this article is available at https://publons.com/publon/10.1002/brb3.70021.

## Supporting information

Supporting Information

## Data Availability

The data that support the findings of this study are available from the corresponding author upon reasonable request.

## References

[brb370021-bib-0001] Araújo, K. M. S. T. , Silva, S. R. A. , Freire, D. A. , Leal, M. C. C. , Marques, A. P. O. , Baptista, R. S. , & Silva, A. L. O. (2021). Correlation between quality of life, depression, satisfaction and functionality of older people with HIV. Revista Brasileira de Enfermagem, 74(Suppl 2), e20201334. 10.1590/0034-7167-2020-1334 34287502

[brb370021-bib-0002] Arends, R. M. , Nelwan, E. J. , Soediro, R. , van Crevel, R. , Alisjahbana, B. , Pohan, H. T. , von Borries, A. K. L. , Schene, A. H. , van der Ven, A. J. A. M. , & Schellekens, A. F. A. (2018). Associations between impulsivity, risk behavior and HIV, HBV, HCV and syphilis seroprevalence among female prisoners in Indonesia: A cross‐sectional study. BioRxiv, 14, 1–13. 10.1101/468694 PMC6377190 30768609

[brb370021-bib-0003] Arora, S. , Peters, A. L. , Burner, E. , Lam, C. N. , & Menchine, M. (2014). Trial to examine text message–Based mhealth in emergency department patients with diabetes (TExT‐MED): A randomized controlled trial. Annals of Emergency Medicine, 63(6), 745–754.e6. 10.1016/j.annemergmed.2013.10.012 24225332

[brb370021-bib-0004] Asrat, B. , Lund, C. , Ambaw, F. , Garman, E. C. , & Schneider, M. (2020). Major depressive disorder and its association with adherence to antiretroviral therapy and quality of life: Cross‐sectional survey of people living with HIV/AIDS in Northwest Ethiopia. BMC Psychiatry, 20(1), 462. 10.1186/s12888-020-02865-w 32972394 PMC7513286

[brb370021-bib-0005] Barratt, E. S. (1985). Impulsiveness subtraits: Arousal and information processing. In C. E. Spence & C. E. Izard (Eds.), Motivation, emotion, and personality (pp. 137–46). Elsevier Science Publishers.

[brb370021-bib-0006] Berry, D. , Blonquist, T. , Hong, F. , Partidge, A. , & Halpenny, B. (2015). Self‐reported adherence to oral cancer therapy: Relationships with symptom distress, depression, and personal characteristics. Patient Preference and Adherence, 9, 1587–1592. 10.2147/PPA.S91534 26604712 PMC4639537

[brb370021-bib-0007] Biederman, J. , Ball, S. W. , Monuteaux, M. C. , Mick, E. , Spencer, T. J. , McCREARY, M. , Cote, M. , & Faraone, S. V. (2008). New insights into the comorbidity between ADHD and major depression in adolescent and young adult females. Journal of the American Academy of Child and Adolescent Psychiatry, 47(4), 426–434. 10.1097/CHI.0b013e31816429d3 18388760

[brb370021-bib-0008] Biney, I. J. K. , Kyei, K. A. , Ganu, V. J. , Kenu, E. , Puplampu, P. , Manortey, S. , & Lartey, M. (2021). Antiretroviral therapy adherence and viral suppression among HIV‐infected adolescents and young adults at a tertiary hospital in Ghana. African Journal of AIDS Research, 20(4), 270–276. 10.2989/16085906.2021.1998783 34905452

[brb370021-bib-0009] Borsboom, D. , & Cramer, A. O. J. (2013). Network analysis: An integrative approach to the structure of psychopathology. Annual Review of Clinical Psychology, 9, 91–121. 10.1146/annurev-clinpsy-050212-185608 23537483

[brb370021-bib-0010] Bramlage, P. , Ketelhut, R. , Fronk, E.‐M. , Wolf, W.‐P. , Smolnik, R. , Zemmrich, C. , & Schmieder, R. E. (2014). Clinical impact of patient adherence to a fixed‐dose combination of olmesartan, amlodipine and hydrochlorothiazide. Clinical Drug Investigation, 34(6), 403–411. 10.1007/s40261-014-0188-z 24719291

[brb370021-bib-0011] Bringmann, L. F. , Elmer, T. , Epskamp, S. , Krause, R. W. , Schoch, D. , Wichers, M. , Wigman, J. T. W. , & Snippe, E. (2019). What do centrality measures measure in psychological networks? Journal of Abnormal Psychology, 128(8), 892–903. 10.1037/abn0000446 31318245

[brb370021-bib-0012] Buchholz, A. , Broekman, T. , & Schippers, G. (2011). Anwendung der ICF in der Suchthilfe am Beispiel des MATE‐ICN. Suchttherapie, 12(1), 14–19. 10.1055/s-0030-1267210

[brb370021-bib-0013] Burnam, M. A. , Bing, E. G. , Morton, S. C. , Sherbourne, C. , Fleishman, J. A. , London, A. S. , Vitiello, B. , Stein, M. , Bozzette, S. A. , & Shapiro, M. F. (2001). Use of mental health and substance abuse treatment services among adults with HIV in the United States. Archives Of General Psychiatry, 58, 729–736. 11483138 10.1001/archpsyc.58.8.729

[brb370021-bib-0014] Cai, H. , Bai, W. , Liu, H. , Chen, X. , Qi, H. , Liu, R. , Cheung, T. , Su, Z. , Lin, J. , Tang, Y. L. , Jackson, T. , Zhang, Q. , & Xiang, Y. T. (2022). Network analysis of depressive and anxiety symptoms in adolescents during the later stage of the COVID‐19 pandemic. Translational Psychiatry, 12(1), 98. 10.1038/s41398-022-01838-9 35273161 PMC8907388

[brb370021-bib-0015] Chander, G. , Himelhoch, S. , & Moore, R. D. (2006). Substance abuse and psychiatric disorders in HIV‐positive patients. Drugs, 66(6), 769–789. 10.2165/00003495-200666060-00004 16706551

[brb370021-bib-0016] Chang, L. , Lim, A. , Lau, E. , & Alicata, D. (2017). Chronic tobacco‐smoking on psychopathological symptoms, impulsivity and cognitive deficits in HIV‐infected individuals. Journal of Neuroimmune Pharmacology, 12(2), 389–401. 10.1007/s11481-017-9728-7 28303534 PMC5529218

[brb370021-bib-0017] Channer, K. S. , Papouchado, M. , James, M. A. , & Rees, J. R. (1985). Anxiety and depression in patients with chest pain referred for exercise testing. Lancet (London, England), 2(8459), 820–823. 10.1016/s0140-6736(85)90805-0 2864541

[brb370021-bib-0018] Ciesla, J. A. , & Roberts, J. E. (2001). Meta‐analysis of the relationship between HIV infection and risk for depressive disorders. American Journal of Psychiatry, 158(5), 725–730. 10.1176/appi.ajp.158.5.725 11329393

[brb370021-bib-0019] Coffey, S. F. , Gudleski, G. D. , Saladin, M. E. , & Brady, K. T. (2003). Impulsivity and rapid discounting of delayed hypothetical rewards in cocaine‐dependent individuals. Experimental and Clinical Psychopharmacology, 11(1), 18–25. 10.1037/1064-1297.11.1.18 12622340

[brb370021-bib-0020] Cooper, V. , Clatworthy, J. , Harding, R. , & Whetham, J. (2017). Measuring quality of life among people living with HIV: A systematic review of reviews. Health and Quality of Life Outcomes, 15(1), 220. 10.1186/s12955-017-0778-6 29141645 PMC5688651

[brb370021-bib-0021] Crystal, S. , Sambamoorthi, U. , Moynihan, P. J. , & McSpiritt, E. (2001). Initiation and continuation of newer antiretroviral treatments among medicaid recipients with AIDS. Journal of General Internal Medicine, 16(12), 850–859. http://www.ncbi.nlm.nih.gov/pubmed/11903765 11903765 10.1111/j.1525-1497.2001.01025.x PMC1495297

[brb370021-bib-0022] Epskamp, S. (2017). Network psychometrics [PhD Thesisis]. University of Amsterdam, Faculty of Social and Behavioural Sciences. https://hdl.handle.net/11245.1/a76273c6‐6abc‐4cc7‐a2e9‐3b5f1ae3c29e

[brb370021-bib-0023] Epskamp, S. , Borsboom, D. , & Fried, E. I. (2018). Estimating psychological networks and their accuracy: A tutorial paper. Behavior Research Methods, 50(1), 195–212. 10.3758/s13428-017-0862-1 28342071 PMC5809547

[brb370021-bib-0024] EuroQol Research Foundation . (2019). EQ‐5D‐5L user guide . EuroQol. https://Euroqol.Org/Publications/User‐Guides; https://euroqol.org/publications/user‐guides

[brb370021-bib-0025] Fino, E. , Jaspal, R. , Lopes, B. , Wignall, L. , & Bloxsom, C. (2021). The sexual risk behaviors scale (SRBS): Development & validation in a university student sample in the UK. Evaluation & the Health Professions, 44(2), 152–160. 10.1177/01632787211003950 33853360 PMC8107449

[brb370021-bib-0026] Fishbein, D. H. , Krupitsky, E. , Flannery, B. A. , Langevin, D. J. , Bobashev, G. , Verbitskaya, E. , Augustine, C. B. , Bolla, K. I. , Zvartau, E. , Schech, B. , Egorova, V. , Bushara, N. , & Tsoy, M. (2007). Neurocognitive characterizations of Russian heroin addicts without a significant history of other drug use. Drug and Alcohol Dependence, 90(1), 25–38. 10.1016/j.drugalcdep.2007.02.015 17382488 PMC1991277

[brb370021-bib-0027] Fried, E. I. , Nesse, R. M. , Zivin, K. , Guille, C. , & Sen, S. (2014). Depression is more than the sum score of its parts: Individual DSM symptoms have different risk factors. Psychological Medicine, 44(10), 2067–2076. 10.1017/S0033291713002900 24289852 PMC4104249

[brb370021-bib-0028] Fried, E. I. , Epskamp, S. , Nesse, R. M. , Tuerlinckx, F. , & Borsboom, D. (2016). What are “good” depression symptoms? Comparing the centrality of DSM and non‐DSM symptoms of depression in a network analysis. Journal of Affective Disorders, 189, 314–320. 10.1016/j.jad.2015.09.005 26458184

[brb370021-bib-0029] Fried, E. I. , & Nesse, R. M. (2015). Depression is not a consistent syndrome: An investigation of unique symptom patterns in the STAR*D study. Journal of Affective Disorders, 172, 96–102. 10.1016/j.jad.2014.10.010 25451401 PMC4397113

[brb370021-bib-0030] Fried, E. I. , van Borkulo, C. D. , Cramer, A. O. J. , Boschloo, L. , Schoevers, R. A. , & Borsboom, D. (2017). Mental disorders as networks of problems: A review of recent insights. Social Psychiatry and Psychiatric Epidemiology, 52(1), 1–10. 10.1007/s00127-016-1319-z 27921134 PMC5226976

[brb370021-bib-0031] Galland, D. , Simioni, N. , Schippers, G. , Broekman, T. , Alaux‐Cantin, S. , Houchi, H. , Naassila, M. , & Rolland, B. (2018). La MATE‐Fr: Introduction à la version française d'un instrument d’évaluation globale en addictologie. Alcoologie et Addictologie, 40(2), 140–148.

[brb370021-bib-0032] Galvan, F. H. , Bing, E. G. , Fleishman, J. A. , London, A. S. , Caetano, R. , Burnam, M. A. , Longshore, D. , Morton, S. C. , Orlando, M. , & Shapiro, M. (2002). The prevalence of alcohol consumption and heavy drinking among people with HIV in the United States: Results from the HIV cost and services utilization study. Journal of Studies on Alcohol, 63(2), 179–186. 10.15288/jsa.2002.63.179 12033694

[brb370021-bib-0033] Gaynes, B. N. , Pence, B. W. , Eron, J. J. , & Miller, W. C. (2008). Prevalence and comorbidity of psychiatric diagnoses based on reference standard in an HIV+ patient population. Psychosomatic Medicine, 70(4), 505–511. 10.1097/PSY.0b013e31816aa0cc 18378865 PMC2900836

[brb370021-bib-0034] Gilliom, M. , & Shaw, D. S. (2004). Codevelopment of externalizing and internalizing problems in early childhood. Development and Psychopathology, 16(2), 313–333. 10.1017/s0954579404044530 15487598

[brb370021-bib-0035] Gonzalez, A. , Barinas, J. , & O'Cleirigh, C. (2011). Substance use: Impact on adherence and HIV medical treatment. Current HIV/AIDS Reports, 8(4), 223–234. 10.1007/s11904-011-0093-5 21858414

[brb370021-bib-0036] Hampel, B. , Kusejko, K. , Kouyos, R. , Böni, J. , Flepp, M. , Stöckle, M. Conen Béguelin, C. , Künzler‐Heule, P. , Nicca, D. , Schmidt, A. J. , Nguyen, H. , Delaloye, J. , Rougemont, M. , Bernasconi, E. , Rauch, A. , Günthard, H. F. , Braun, D. L. , & Fehr, J. , (2020). Chemsex drugs on the rise: A longitudinal analysis of the Swiss HIV cohort study from 2007 to 2017. HIV Medicine, 21(4), 228–239. 10.1111/hiv.12821 31849182

[brb370021-bib-0037] Han, S. , Zhang, Y. , Yang, X. , Li, K. , Zhang, L. , Shao, Y. , Ma, J. , Hu, Y. , Zhu, Z. , Zhang, Y. , & Wang, Z. (2023). Exploring core mental health symptoms among persons living with HIV: A network analysis. Frontiers in Psychiatry, 14, 1081867. 10.3389/fpsyt.2023.1081867 36741117 PMC9895861

[brb370021-bib-0038] Hell, M. E. , Andersen, K. , & Nielsen, A. S. (2018). Is the Danish version of MATE feasible? A pilot study on feasibility and adequacy. Journal of Dual Diagnosis, 14(1), 14–20. 10.1080/15504263.2017.1386810 29053436

[brb370021-bib-0039] Helleberg, M. , Afzal, S. , Kronborg, G. , Larsen, C. S. , Pedersen, G. , Pedersen, C. , Gerstoft, J. , Nordestgaard, B. G. , & Obel, N. (2013). Mortality attributable to smoking among HIV‐1—Infected individuals : A nationwide, population‐based cohort study. Clinical Infectious Diseases, 56, 727–734. 10.1093/cid/cis933 23254417

[brb370021-bib-0040] Herdman, M. , Gudex, C. , Lloyd, A. , Janssen, M. , Kind, P. , Parkin, D. , Bonsel, G. , & Badia, X. (2011). Development and preliminary testing of the new five‐level version of EQ‐5D (EQ‐5D‐5L). Quality of Life Research, 20(10), 1727–1736. 10.1007/S11136-011-9903-X 21479777 PMC3220807

[brb370021-bib-0041] Himelhoch, S. , Josephs, J. S. , Chander, G. , Korthuis, P. T. , & Gebo, K. A. (2009). Use of outpatient mental health services and psychotropic medications among HIV‐infected patients in a multisite, multistate study. General Hospital Psychiatry, 31(6), 538–545. 10.1016/j.genhosppsych.2009.05.009 19892212 PMC3144858

[brb370021-bib-0042] Jain, D. , Kumar, Y. M. P. , Katyal, V. K. , Jain, P. , Kumar, J. P. , & Singh, S. (2021). Study of quality of life and depression in people living with HIV/AIDS in India. AIDS Reviews, 23(4), 186–195. 10.24875/AIDSRev.20000114 34980927

[brb370021-bib-0043] Kessler, R. C. (1994). Lifetime and 12‐month prevalence of DSM‐III‐R psychiatric disorders in the United States. Archives of General Psychiatry, 51(1), 8–19. 10.1001/archpsyc.1994.03950010008002 8279933

[brb370021-bib-0044] Kjome, K. , Lane, S. , Schmitz, J. , Green, C. , Ma, L. , Prasla, I. , Swann, A. C. , & Moeller, G. (2010). Relationship between impulsivity and decision‐making in cocaine dependence. Psychiatry Research, 178(2), 299–304. 10.1016/j.psychres.2009.11.024 20478631 PMC2904056

[brb370021-bib-0045] Kreek, M. J. , Nielsen, D. A. , Butelman, E. R. , & LaForge, K. S. (2005). Genetic influences on impulsivity, risk taking, stress responsivity and vulnerability to drug abuse and addiction. Nature Neuroscience, 8(11), 1450–1457. 10.1038/nn1583 16251987

[brb370021-bib-0046] Lamers, F. , van Oppen, P. , Comijs, H. C. , Smit, J. H. , Spinhoven, P. , van Balkom, A. J. L. M. , Nolen, W. A. , Zitman, F. G. , Beekman, A. T. , & Penninx, B. W. J. H. (2011). Comorbidity patterns of anxiety and depressive disorders in a large cohort study. The Journal of Clinical Psychiatry, 72(03), 341–348. 10.4088/JCP.10m06176blu 21294994

[brb370021-bib-0047] Lewis, G. (1991). Observer bias in the assessment of anxiety and depression. Social Psychiatry and Psychiatric Epidemiology, 26(6), 265–272. 10.1007/BF00789218 1792557

[brb370021-bib-0048] López‐Toro, E. , Wolf, C. J. H. , González, R. A. , van den Brink, W. , Schellekens, A. , & Vélez‐Pastrana, M. C. (2022). Network analysis of DSM symptoms of substance use disorders and frequently co‐occurring mental disorders in patients with substance use disorder who seek treatment. Journal of Clinical Medicine, 11(10), 2883. 10.3390/jcm11102883 35629008 PMC9145186

[brb370021-bib-0049] Lucas, G. M. , Cheever, L. W. , Chaisson, R. E. , & Moore, R. D. (2001). Detrimental effects of continued illicit drug use on the treatment of HIV‐1 infection. JAIDS Journal of Acquired Immune Deficiency Syndromes, 27(3), 251–259. 10.1097/00126334-200107010-00006 11464144

[brb370021-bib-0050] Lucas, G. M. , Gebo, K. A. , Chaisson, R. E. , & Moore, R. D. (2002). Longitudinal assessment of the effects of drug and alcohol abuse on HIV‐1 treatment outcomes in an urban clinic. AIDS, 16(5), 767–774. 10.1097/00002030-200203290-00012 11964533

[brb370021-bib-0051] Luseno, W. K. , Field, S. H. , Iritani, B. J. , Odongo, F. S. , Kwaro, D. , Amek, N. O. , & Rennie, S. (2021). Pathways to depression and poor quality of life among adolescents in Western Kenya: Role of anticipated HIV stigma, HIV risk perception, and sexual behaviors. AIDS and Behavior, 25(5), 1423–1437. 10.1007/s10461-020-02980-5 32737818 PMC7855560

[brb370021-bib-0052] Malta, M. , Strathdee, S. A. , Magnanini, M. M. F. , & Bastos, F. I. (2008). Adherence to antiretroviral therapy for human immunodeficiency virus/acquired immune deficiency syndrome among drug users: A systematic review. Addiction, 103(8), 1242–1257. 10.1111/j.1360-0443.2008.02269.x 18855813

[brb370021-bib-0053] Mayston, R. , Kinyanda, E. , Chishinga, N. , Prince, M. , & Patel, V. (2012). Mental disorder and the outcome of HIV/AIDS in low‐income and middle‐income countries: A systematic review. AIDS, 26(Suppl 2), 117–135. 10.1097/QAD.0b013e32835bde0f 23303434

[brb370021-bib-0054] Meade, C. S. , & Sikkema, K. J. (2005). HIV risk behavior among adults with severe mental illness: A systematic review. Clinical Psychology Review, 25(4), 433–457. 10.1016/j.cpr.2005.02.001 15914265

[brb370021-bib-0055] Meeder, E. , Matzaraki, V. , Vadaq, N. , van de Wijer, L. , van der Ven, A. , & Schellekens, A. (2021). Unbiased metabolomics links fatty acid pathways to psychiatric symptoms in people living with HIV. Journal of Clinical Medicine, 10(23), 5466. 10.3390/jcm10235466 34884168 PMC8658345

[brb370021-bib-0056] Mitchell, J. M. , Fields, H. L. , D'Esposito, M. , & Boettiger, C. A. (2005). Impulsive responding in alcoholics. Alcoholism: Clinical and Experimental Research, 29(12), 2158–2169. 10.1097/01.alc.0000191755.63639.4a 16385186

[brb370021-bib-0057] Moon, S. J. , Lee, W. Y. , Hwang, J. S. , Hong, Y. P. , & Morisky, D. E. (2017). Accuracy of a screening tool for medication adherence: A systematic review and meta‐analysis of the Morisky Medication Adherence Scale‐8. PLoS ONE, 12(11), 1–18. 10.1371/journal.pone.0187139 PMC5667769 29095870

[brb370021-bib-0058] Moorey, S. , Greer, S. , Watson, M. , Gorman, C. , Rowden, L. , Tunmore, R. , Robertson, B. , & Bliss, J. (1991). The factor structure and factor stability of the hospital anxiety and depression scale in patients with cancer. British Journal of Psychiatry, 158(2), 255–259. 10.1192/bjp.158.2.255 1812841

[brb370021-bib-0059] Morisky, D. E. , Ang, A. , Krousel‐Wood, M. , & Ward, H. J. (2008). Predictive validity of a medication adherence measure in an outpatient setting. The Journal of Clinical Hypertension, 10(5), 348–354. 10.1111/j.1751-7176.2008.07572.x 18453793 PMC2562622

[brb370021-bib-0060] Morisky, D. E. , Green, L. W. , & Levine, D. M. (1986). Concurrent and predictive validity of a self‐reported measure of medication adherence. Medical Care, 24(1), 67–74. 10.1097/00005650-198601000-00007 3945130

[brb370021-bib-0061] Okimat, P. , Akena, D. , Opio, D. , Mutabazi, T. , Sendaula, E. , Semitala, F. C. , Kalyango, J. N. , & Karamagi, C. A. (2022). Screening PLHIV for depression using PHQs: A RCT comparing non‐selective with selective screening strategy within a primary health care facility in Uganda. PloS ONE, 17(6), e0270175. 10.1371/journal.pone.0270175 35767586 PMC9242435

[brb370021-bib-0062] Oudejans, S. , De Weert‐Van Oene, G. , Spits, M. , De Wildt, W. , Merkx, M. , Dekker, J. , Visch, I. , & Goudriaan, A. (2020). A self‐reported version of the measurements in the addictions for triage and evaluation‐Q: Concurrent validity with the MATE 2.1. European Addiction Research, 26(1), 20–27. 10.1159/000503625 31639811 PMC6979419

[brb370021-bib-0063] Park, L. G. , Howie‐Esquivel, J. , Chung, M. L. , & Dracup, K. (2014). A text messaging intervention to promote medication adherence for patients with coronary heart disease: A randomized controlled trial. Patient Education and Counseling, 94(2), 261–268. 10.1016/j.pec.2013.10.027 24321403

[brb370021-bib-0064] Patton, J. H. , Stanford, M. S. , & Barratt, E. S. (1995). Factor structure of the Barratt impulsiveness scale. Journal of Clinical Psychology, 51(6), 768–774. 10.1002/1097-4679(199511)51:6<768::AID-JCLP2270510607>3.0.CO;2-1 8778124

[brb370021-bib-0065] Powell, V. , Martin, J. , Thapar, A. , Rice, F. , & Anney, R. J. L. (2021). Investigating regions of shared genetic variation in attention deficit/hyperactivity disorder and major depressive disorder: A GWAS meta‐analysis. Scientific Reports, 11(1), 7353. 10.1038/s41598-021-86802-1 33795730 PMC8016853

[brb370021-bib-0066] Reda, A. A. (2011). Reliability and validity of the Ethiopian version of the hospital anxiety and depression scale (HADS) in HIV infected patients. PLoS ONE, 6(1), 1–6. 10.1371/journal.pone.0016049 PMC3026786 21283565

[brb370021-bib-0067] Reise, S. P. , Moore, T. M. , Sabb, F. W. , Brown, A. K. , & London, E. D. (2013). The Barratt impulsiveness scale–11: Reassessment of its structure in a community sample. Psychological Assessment, 25(2), 631–642. 10.1037/a0032161 23544402 PMC3805371

[brb370021-bib-0068] Samet, J. H. , Phillips, S. J. , Horton, N. J. , Traphagen, E. T. , & Freedberg, K. A. (2004). Detecting alcohol problems in HIV‐infected patients: Use of the CAGE questionnaire. AIDS Research and Human Retroviruses, 20(2), 151–155. 10.1089/088922204773004860 15018702

[brb370021-bib-0069] Sebit, M. , Tombe, M. , Siziya, S. , Balus, S. , Nkomo, S. , & Maramba, P. (2004). Prevalence of HIV/AIDS and psychiatric disorders and their related risk factors among adults in Epworth, Zimbabwe. East African Medical Journal, 80(10), 503–512. 10.4314/eamj.v80i10.8752 15250622

[brb370021-bib-0070] Shaffer, D. N. , Njeri, R. , Justice, A. C. , Odero, W. W. , & Tierney, W. M. (2004). Alcohol abuse among patients with and without HIV infection attending public clinics in Western Kenya. East African Medical Journal, 81(11), 594–598. http://www.ncbi.nlm.nih.gov/pubmed/15868970 15868970

[brb370021-bib-0071] Spinhoven, P. , Ormel, J. , Sloekers, P. P. , Kempen, G. I. , Speckens, A. E. , & Van Hemert, A. M. (1997). A validation study of the hospital anxiety and depression scale (HADS) in different groups of Dutch subjects. Psychological Medicine, 27(2), 363–370. 10.1017/s0033291796004382 9089829

[brb370021-bib-0072] Stanford, M. S. , Mathias, C. W. , Dougherty, D. M. , Lake, S. L. , Anderson, N. E. , & Patton, J. H. (2009). Fifty years of the Barratt impulsiveness scale: An update and review. Personality and Individual Differences, 47(5), 385–395. 10.1016/j.paid.2009.04.008

[brb370021-bib-0073] Tran, B. , Ohinmaa, A. , & Nguyen, L. (2012). Quality of life profile and psychometric properties of the EQ‐5D‐5L in HIV/AIDS patients. Health and Quality of Life Outcomes, 10(1), 132. 10.1186/1477-7525-10-132 23116130 PMC3541089

[brb370021-bib-0074] Trimbos Institute . (2022). Nationale drug monitor . Trimbos Institute. https://Www.Nationaledrugmonitor.Nl/; https://www.nationaledrugmonitor.nl/ecstasy‐gebruik‐algemene‐bevolking/

[brb370021-bib-0075] Van Borkulo, C. D. , Borsboom, D. , Epskamp, S. , Blanken, T. F. , Boschloo, L. , Schoevers, R. A. , & Waldorp, L. J. (2014). A new method for constructing networks from binary data. Scientific Reports, 4, 1–10. 10.1038/srep05918 PMC4118196 25082149

[brb370021-bib-0076] Van de Wijer, L. , van der Heijden, W. , van Verseveld, M. , Netea, M. , de Mast, Q. , Schellekens, A. , & van der Ven, A. (2021). Substance use, unlike dolutegravir, is associated with mood symptoms in people living with HIV. AIDS and Behavior, 25(12), 4094–4101. 10.1007/S10461-021-03272-2 33903997 PMC8602138

[brb370021-bib-0077] Verdejo‐García, A. J. , Perales, J. C. , & Pérez‐García, M. (2007). Cognitive impulsivity in cocaine and heroin polysubstance abusers. Addictive Behaviors, 32(5), 950–966. 10.1016/j.addbeh.2006.06.032 16876962

[brb370021-bib-0078] Von den Hoff, D. W. , Berden, F. A. C. , Atsma, F. , Schellekens, A. F. A. , & Drenth, J. P. H. (2022). Against all odds? Addiction history associated with better viral hepatitis care: A Dutch nationwide claims data study. Journal of Clinical Medicine, 11(4), 1146. 10.3390/jcm11041146 35207419 PMC8878485

[brb370021-bib-0079] Vos, W. A. J. W. , Groenendijk, A. L. , Blaauw, M. J. T. , van Eekeren, L. E. , Navas, A. , Cleophas, M. C. P. , Vadaq, N. , Matzaraki, V. , Dos Santos, J. C. , Meeder, E. M. G. , Fröberg, J. , Weijers, G. , Zhang, Y. , Fu, J. , Ter Horst, R. , Bock, C. , Knoll, R. , Aschenbrenner, A. C. , Schultze, J. , … van der Ven, A. J. A. M. (2022). The 2000HIV study: Design, multi‐omics methods and participant characteristics. Frontiers in Immunology, 13, 982746. https://www.frontiersin.org/articles/10.3389/fimmu.2022.982746 36605197 10.3389/fimmu.2022.982746 PMC9809279

[brb370021-bib-0080] Wen, H. , Zhu, Z. , Hu, T. , Li, C. , Jiang, T. , Li, L. , Zhang, L. , Fu, Y. , Han, S. , Wu, B. , & Hu, Y. (2023). Unraveling the central and bridge psychological symptoms of people living with HIV: A network analysis. Frontiers in Public Health, 10, 1024436. 10.3389/fpubh.2022.1024436 36684950 PMC9846149

[brb370021-bib-0081] Willoughby, M. , Weinberger, A. H. , Shuter, J. , & Seng, E. K. (2021). Pain and medication adherence in adult cigarette smokers living with HIV: A cross‐sectional observational study. AIDS Care, 33(11), 1422–1429. 10.1080/09540121.2020.1849530 33233919 PMC8144233

[brb370021-bib-0082] Wouters, E. , Booysen Fle, R. , Ponnet, K. , & Baron van Loon, F. (2012). Wording effects and the factor structure of the hospital anxiety & depression scale in HIV/AIDS patients on antiretroviral treatment in South Africa. PLoS ONE, 7(4), e34881. 10.1371/journal.pone.0034881 22536338 PMC3335020

[brb370021-bib-0083] Xiaowen, W. , Guangping, G. , Ling, Z. , Jiarui, Z. , Xiumin, L. , Zhaoqin, L. , Hongzhuan, L. , Yuyan, Y. , Liyuan, Y. , & Lin, L. (2018). Depression and anxiety mediate perceived social support to predict health‐related quality of life in pregnant women living with HIV. AIDS Care, 30(9), 1147–1155. 10.1080/09540121.2018.1456640 29607666

[brb370021-bib-0084] Zhu, Z. , Guo, M. , Dong, T. , Han, S. , Hu, Y. , & Wu, B. (2022). Assessing psychological symptom networks related to HIV‐positive duration among people living with HIV: A network analysis. AIDS Care, 34(6), 725–733. 10.1080/09540121.2021.1929815 34043459

[brb370021-bib-0085] Zigmond, A. S. , & Snalth, R. P. (1983). The hospital anxiety and depression scale. Acta psychiatr. scand. Acta Psychiatrica Scandinavica, 67(6), 361–370. https://www.ncbi.nlm.nih.gov/pubmed/6880820 6880820 10.1111/j.1600-0447.1983.tb09716.x

[brb370021-bib-0086] Zisner, A. , & Beauchaine, T. P. (2016). Neural substrates of trait impulsivity, anhedonia, and irritability: Mechanisms of heterotypic comorbidity between externalizing disorders and unipolar depression. Development and Psychopathology, 28, (4 part 1), 1177–1208. 10.1017/S0954579416000754 27739396

