# Supplementary material for: Mental health and its consequences in people living with HIV: A network approach
Source: Brain Behav. 2024 Oct 20;14(10):e70021. doi: 10.1002/brb3.70021 (PMC11491311; doi:10.1002/brb3.70021)
Supplement: Supplementary file 1 — Supporting Information [file BRB3-14-e70021-s001.docx]

**Supplementary Table 1.** Internal consistency of self-questionnaires within the 2000HIV sample

| **Questionnaire** | **Cronbachs α** | **Questionnaire** | **Cronbachs α** |
| --- | --- | --- | --- |
| HADS |  | BIS-11 |  |
| Total scale | 0.90 | Total scale | 0.77 |
| Depression subscale | 0.82 | Motor subscale | 0.58 |
| Anxiety subscale | 0.87 | Attentional subscale | 0.60 |
| MMAS-8 | 0.62 | Nonplanning subscale | 0.66 |
| EQ-5D-5L | 0.76 |  |  |

**Supplementary Table 2.** Variable distribution before and after dichotomisation

|  | **Before dichotomisation** | | | **After dichotomisation** | | |
| --- | --- | --- | --- | --- | --- | --- |
| **Variable** | **Level** | **Frequency** | **Proportion (%)** | **Level** | **Frequency** | **Proportion (%)** |
| **B2** | 1 | 598 | 37.03 | 0 | 598 | 37.03 |
|  | 2 | 782 | 48.42 | 1 | 1017 | 62.97 |
|  | 3 | 196 | 12.14 |  |  |  |
|  | 4 | 39 | 2.41 |  |  |  |
| **B3** | 1 | 172 | 10.65 | 0 | 172 | 10.65 |
|  | 2 | 673 | 41.67 | 1 | 1443 | 89.35 |
|  | 3 | 571 | 35.36 |  |  |  |
|  | 4 | 199 | 12.32 |  |  |  |
| **B4** | 1 | 291 | 18.02 | 0 | 291 | 18.02 |
|  | 2 | 542 | 33.56 | 1 | 1324 | 81.98 |
|  | 3 | 536 | 33.19 |  |  |  |
|  | 4 | 246 | 15.23 |  |  |  |
| **B16** | 1 | 1097 | 67.93 | 0 | 1097 | 67.93 |
|  | 2 | 427 | 26.44 | 1 | 518 | 32.07 |
|  | 3 | 80 | 4.95 |  |  |  |
|  | 4 | 11 | 0.68 |  |  |  |
| **B17** | 1 | 509 | 31.52 | 0 | 509 | 31.52 |
|  | 2 | 874 | 54.12 | 1 | 1106 | 68.48 |
|  | 3 | 198 | 12.26 |  |  |  |
|  | 4 | 34 | 2.11 |  |  |  |
| **B19** | 1 | 534 | 33.07 | 0 | 534 | 33.07 |
|  | 2 | 909 | 56.28 | 1 | 1081 | 66.93 |
|  | 3 | 141 | 8.73 |  |  |  |
|  | 4 | 31 | 1.92 |  |  |  |
| **B21** | 1 | 1179 | 73.00 | 0 | 1179 | 73.00 |
|  | 2 | 365 | 22.60 | 1 | 436 | 27.00 |
|  | 3 | 65 | 4.02 |  |  |  |
|  | 4 | 6 | 0.37 |  |  |  |
| **B22** | 1 | 705 | 43.65 | 0 | 705 | 43.65 |
|  | 2 | 762 | 47.18 | 1 | 910 | 56.35 |
|  | 3 | 126 | 7.80 |  |  |  |
|  | 4 | 22 | 1.36 |  |  |  |
| **B23** | 1 | 545 | 33.75 | 0 | 545 | 33.75 |
|  | 2 | 630 | 39.01 | 1 | 1070 | 66.25 |
|  | 3 | 304 | 18.82 |  |  |  |
|  | 4 | 136 | 8.42 |  |  |  |
| **B25** | 1 | 1148 | 71.08 | 0 | 1148 | 71.08 |
|  | 2 | 369 | 22.85 | 1 | 467 | 28.92 |
|  | 3 | 75 | 4.64 |  |  |  |
|  | 4 | 23 | 1.42 |  |  |  |
| **B30** | 1 | 241 | 14.92 | 0 | 241 | 14.92 |
|  | 2 | 512 | 31.70 | 1 | 1374 | 85.08 |
|  | 3 | 646 | 40.00 |  |  |  |
|  | 4 | 216 | 13.37 |  |  |  |
| **B1** | 1 | 492 | 30.46 | 0 | 492 | 30.46 |
|  | 2 | 605 | 37.46 | 1 | 1123 | 69.54 |
|  | 3 | 423 | 26.19 |  |  |  |
|  | 4 | 95 | 5.88 |  |  |  |
| **B7** | 1 | 268 | 16.59 | 0 | 268 | 16.59 |
|  | 2 | 487 | 30.15 | 1 | 1347 | 83.41 |
|  | 3 | 605 | 37.46 |  |  |  |
|  | 4 | 255 | 15.79 |  |  |  |
| **B8** | 1 | 810 | 50.15 | 0 | 810 | 50.15 |
|  | 2 | 620 | 38.39 | 1 | 805 | 49.85 |
|  | 3 | 164 | 10.15 |  |  |  |
|  | 4 | 21 | 1.30 |  |  |  |
| **B10** | 1 | 498 | 30.84 | 0 | 498 | 30.84 |
|  | 2 | 482 | 29.85 | 1 | 1117 | 69.16 |
|  | 3 | 415 | 25.70 |  |  |  |
|  | 4 | 220 | 13.62 |  |  |  |
| **B12** | 1 | 539 | 33.37 | 0 | 539 | 33.37 |
|  | 2 | 762 | 47.18 | 1 | 1076 | 66.63 |
|  | 3 | 291 | 18.02 |  |  |  |
|  | 4 | 23 | 1.42 |  |  |  |
| **B13** | 1 | 1047 | 64.83 | 0 | 1047 | 64.83 |
|  | 2 | 311 | 19.26 | 1 | 568 | 35.17 |
|  | 3 | 119 | 7.37 |  |  |  |
|  | 4 | 138 | 8.54 |  |  |  |
| **B14** | 1 | 628 | 38.89 | 0 | 628 | 38.89 |
|  | 2 | 816 | 50.53 | 1 | 987 | 61.11 |
|  | 3 | 130 | 8.05 |  |  |  |
|  | 4 | 41 | 2.54 |  |  |  |
| **B15** | 1 | 269 | 16.66 | 0 | 269 | 16.66 |
|  | 2 | 560 | 34.67 | 1 | 1346 | 83.34 |
|  | 3 | 588 | 36.41 |  |  |  |
|  | 4 | 198 | 12.26 |  |  |  |
| **B18** | 1 | 850 | 52.63 | 0 | 850 | 52.63 |
|  | 2 | 612 | 37.89 | 1 | 765 | 47.37 |
|  | 3 | 131 | 8.11 |  |  |  |
|  | 4 | 22 | 1.36 |  |  |  |
| **B27** | 1 | 251 | 15.54 | 0 | 251 | 15.54 |
|  | 2 | 734 | 45.45 | 1 | 1364 | 84.46 |
|  | 3 | 464 | 28.73 |  |  |  |
|  | 4 | 166 | 10.28 |  |  |  |
| **B29** | 1 | 115 | 7.12 | 0 | 115 | 7.12 |
|  | 2 | 303 | 18.76 | 1 | 1500 | 92.88 |
|  | 3 | 643 | 39.81 |  |  |  |
|  | 4 | 554 | 34.30 |  |  |  |
| **B5** | 1 | 600 | 37.15 | 0 | 600 | 37.15 |
|  | 2 | 777 | 48.11 | 1 | 1015 | 62.85 |
|  | 3 | 169 | 10.46 |  |  |  |
|  | 4 | 69 | 4.27 |  |  |  |
| **B6** | 1 | 543 | 33.62 | 0 | 543 | 33.62 |
|  | 2 | 651 | 40.31 | 1 | 1072 | 66.38 |
|  | 3 | 310 | 19.20 |  |  |  |
|  | 4 | 111 | 6.87 |  |  |  |
| **B9** | 1 | 466 | 28.85 | 0 | 466 | 28.85 |
|  | 2 | 681 | 42.17 | 1 | 1149 | 71.15 |
|  | 3 | 379 | 23.47 |  |  |  |
|  | 4 | 89 | 5.51 |  |  |  |
| **B11** | 1 | 912 | 56.47 | 0 | 912 | 56.47 |
|  | 2 | 391 | 24.21 | 1 | 703 | 43.53 |
|  | 3 | 166 | 10.28 |  |  |  |
|  | 4 | 146 | 9.04 |  |  |  |
| **B20** | 1 | 43 | 2.66 | 0 | 43 | 2.66 |
|  | 2 | 217 | 13.44 | 1 | 1572 | 97.34 |
|  | 3 | 1038 | 64.27 |  |  |  |
|  | 4 | 317 | 19.63 |  |  |  |
| **B24** | 1 | 1159 | 71.76 | 0 | 1159 | 71.76 |
|  | 2 | 393 | 24.33 | 1 | 456 | 28.24 |
|  | 3 | 46 | 2.85 |  |  |  |
|  | 4 | 17 | 1.05 |  |  |  |
| **B26** | 1 | 372 | 23.03 | 0 | 372 | 23.03 |
|  | 2 | 896 | 55.48 | 1 | 1243 | 76.97 |
|  | 3 | 287 | 17.77 |  |  |  |
|  | 4 | 60 | 3.72 |  |  |  |
| **B28** | 1 | 807 | 49.97 | 0 | 807 | 49.97 |
|  | 2 | 598 | 37.03 | 1 | 808 | 50.03 |
|  | 3 | 155 | 9.60 |  |  |  |
|  | 4 | 55 | 3.41 |  |  |  |
| **H1** | 0 | 467 | 28.92 | 0 | 467 | 28.92 |
|  | 1 | 896 | 55.48 | 1 | 1148 | 71.08 |
|  | 2 | 190 | 11.76 |  |  |  |
|  | 3 | 62 | 3.84 |  |  |  |
| **H3** | 0 | 916 | 56.72 | 0 | 916 | 56.72 |
|  | 1 | 393 | 24.33 | 1 | 699 | 43.28 |
|  | 2 | 247 | 15.29 |  |  |  |
|  | 3 | 59 | 3.65 |  |  |  |
| **H5** | 0 | 786 | 48.67 | 0 | 786 | 48.67 |
|  | 1 | 523 | 32.38 | 1 | 829 | 51.33 |
|  | 2 | 248 | 15.36 |  |  |  |
|  | 3 | 58 | 3.59 |  |  |  |
| **H7** | 0 | 648 | 40.12 | 0 | 648 | 40.12 |
|  | 1 | 627 | 38.82 | 1 | 967 | 59.88 |
|  | 2 | 298 | 18.45 |  |  |  |
|  | 3 | 42 | 2.60 |  |  |  |
| **H9** | 0 | 1125 | 69.66 | 0 | 1125 | 69.66 |
|  | 1 | 433 | 26.81 | 1 | 490 | 30.34 |
|  | 2 | 40 | 2.48 |  |  |  |
|  | 3 | 17 | 1.05 |  |  |  |
| **H11** | 0 | 742 | 45.94 | 0 | 742 | 45.94 |
|  | 1 | 629 | 38.95 | 1 | 873 | 54.06 |
|  | 2 | 206 | 12.76 |  |  |  |
|  | 3 | 38 | 2.35 |  |  |  |
| **H13** | 0 | 1082 | 67.00 | 0 | 1082 | 67.00 |
|  | 1 | 420 | 26.01 | 1 | 533 | 33.00 |
|  | 2 | 91 | 5.63 |  |  |  |
|  | 3 | 22 | 1.36 |  |  |  |
| **H2** | 0 | 856 | 53.00 | 0 | 856 | 53.00 |
|  | 1 | 566 | 35.05 | 1 | 759 | 47.00 |
|  | 2 | 160 | 9.91 |  |  |  |
|  | 3 | 33 | 2.04 |  |  |  |
| **H4** | 0 | 1061 | 65.70 | 0 | 1061 | 65.70 |
|  | 1 | 442 | 27.37 | 1 | 554 | 34.30 |
|  | 2 | 93 | 5.76 |  |  |  |
|  | 3 | 19 | 1.18 |  |  |  |
| **H6** | 0 | 958 | 59.32 | 0 | 958 | 59.32 |
|  | 1 | 434 | 26.87 | 1 | 657 | 40.68 |
|  | 2 | 160 | 9.91 |  |  |  |
|  | 3 | 63 | 3.90 |  |  |  |
| **H8** | 0 | 607 | 37.59 | 0 | 607 | 37.59 |
|  | 1 | 767 | 47.49 | 1 | 1008 | 62.41 |
|  | 2 | 191 | 11.83 |  |  |  |
|  | 3 | 50 | 3.10 |  |  |  |
| **H10** | 0 | 1072 | 66.38 | 0 | 1072 | 66.38 |
|  | 1 | 357 | 22.11 | 1 | 543 | 33.62 |
|  | 2 | 159 | 9.85 |  |  |  |
|  | 3 | 27 | 1.67 |  |  |  |
| **H12** | 0 | 904 | 55.98 | 0 | 904 | 55.98 |
|  | 1 | 448 | 27.74 | 1 | 711 | 44.02 |
|  | 2 | 204 | 12.63 |  |  |  |
|  | 3 | 59 | 3.65 |  |  |  |
| **H14** | 0 | 1177 | 72.88 | 0 | 1177 | 72.88 |
|  | 1 | 321 | 19.88 | 1 | 438 | 27.12 |
|  | 2 | 75 | 4.64 |  |  |  |
|  | 3 | 42 | 2.60 |  |  |  |
| **QM** | 0 | 1310 | 81.11 | 0 | 1310 | 81.11 |
|  | 1 | 178 | 11.02 | 1 | 305 | 18.89 |
|  | 2 | 98 | 6.07 |  |  |  |
|  | 3 | 28 | 1.73 |  |  |  |
|  | 4 | 1 | 0.06 |  |  |  |
| **QS** | 0 | 1572 | 97.34 | 0 | 1572 | 97.34 |
|  | 1 | 32 | 1.98 | 1 | 43 | 2.66 |
|  | 2 | 11 | 0.68 |  |  |  |
|  | 3 | 0 | 0.00 |  |  |  |
|  | 4 | 0 | 0.00 |  |  |  |
| **QA** | 0 | 1340 | 82.97 | 0 | 1340 | 82.97 |
|  | 1 | 185 | 11.46 | 1 | 275 | 17.03 |
|  | 2 | 64 | 3.96 |  |  |  |
|  | 3 | 21 | 1.30 |  |  |  |
|  | 4 | 5 | 0.31 |  |  |  |
| **QP** | 0 | 908 | 56.22 | 0 | 908 | 56.22 |
|  | 1 | 484 | 29.97 | 1 | 707 | 43.78 |
|  | 2 | 179 | 11.08 |  |  |  |
|  | 3 | 40 | 2.48 |  |  |  |
|  | 4 | 4 | 0.25 |  |  |  |
| **QV** | 0 | 1 | 33.33 | 0 | 420 | 26.01 |
|  | 1 | 2 | 66.67 | 1 | 1195 | 73.99 |
|  | 2 | 0 | 0.00 |  |  |  |
|  | 3 | 0 | 0.00 |  |  |  |
|  | 4 | 0 | 0.00 |  |  |  |
| **T8** | 0 | 874 | 54.12 | 0 | 874 | 54.12 |
|  | 1 | 663 | 41.05 | 1 | 741 | 45.88 |
|  | 2 | 71 | 4.40 |  |  |  |
|  | 3 | 4 | 0.25 |  |  |  |
|  | 4 | 3 | 0.19 |  |  |  |

**Supplementary Table 3.** Network edge weights (i.e. connection strengths).

|  | **B2** | **B3** | **B4** | **B16** | **B17** | **B19** | **B21** | **B22** | **B23** |
| --- | --- | --- | --- | --- | --- | --- | --- | --- | --- |
| **B2** | 0 | 0.6461 | 0.1503 | 0.2439 | 0.5950 | 0.4901 | 0 | 0.2170 | 0 |
| **B3** | 0.6461 | 0 | 0.2869 | 0 | 0 | 0 | 0 | 0 | 0 |
| **B4** | 0.1503 | 0.2869 | 0 | 0 | 0 | 0 | 0 | 0 | 0 |
| **B16** | 0.2439 | 0 | 0 | 0 | 0 | 0 | 0.9358 | 0 | 0 |
| **B17** | 0.5950 | 0 | 0 | 0 | 0 | 1.9298 | 0 | 0.3134 | 0 |
| **B19** | 0.4901 | 0 | 0 | 0 | 1.9298 | 0 | 0 | 0.6042 | 0 |
| **B21** | 0 | 0 | 0 | 0.9358 | 0 | 0 | 0 | 0 | 0 |
| **B22** | 0.2170 | 0 | 0 | 0 | 0.3134 | 0.6042 | 0 | 0 | 0 |
| **B23** | 0 | 0 | 0 | 0 | 0 | 0 | 0 | 0 | 0 |
| **B25** | 0 | 0 | 0 | 0 | 0 | 0 | 0 | 0.3137 | 0 |
| **B30** | 0 | 0 | 0 | 0 | 0 | 0 | 0 | 0 | 0 |
| **B1** | 0.5512 | 0 | 0 | 0 | 0.2026 | 0 | 0 | 0 | 0 |
| **B7** | 0.2738 | 0 | 0 | 0 | 0 | 0 | 0 | 0 | 0 |
| **B8** | 0 | 0 | 0 | 0 | 0 | 0 | 0 | 0 | 0 |
| **B10** | 0 | 0 | 0 | 0 | 0 | 0 | 0 | 0.0911 | 0 |
| **B12** | 0.4236 | 0 | 0 | 0 | 0 | 0.2260 | 0 | 0.0774 | 0 |
| **B13** | 0 | 0 | 0 | 0.4247 | 0 | 0 | 0 | 0 | 0 |
| **B14** | 0.5079 | 0 | 0 | 0 | 0.7648 | 0.3529 | 0 | 0 | 0 |
| **B15** | 0 | 0 | 0 | 0 | 0 | 0 | 0 | 0 | 0 |
| **B18** | 0 | 0 | 0 | 0 | 0.2246 | 0 | 0 | 0.0463 | 0.1960 |
| **B27** | 0 | 0 | 0 | 0 | 0 | 0.0828 | 0 | 0 | 0.1372 |
| **B29** | 0 | 0 | 0 | 0 | 0 | 0 | 0 | 0 | 0 |
| **B5** | 0.5215 | 0 | 0 | 0 | 0.1368 | 0 | 0 | 0 | 0.2050 |
| **B6** | 0 | 0 | 0 | 0 | 0 | 0 | 0 | 0 | 0 |
| **B9** | 0 | 0 | 0 | 0 | 0 | 0 | 0 | 0 | 0.1725 |
| **B11** | 0 | 0 | 0 | 0 | 0 | 0 | 0 | 0 | 0 |
| **B20** | 0 | 0 | 0 | 0 | 0 | 0 | 0 | 0 | 0 |
| **B24** | 0 | 0 | 0 | 0.4176 | 0 | 0.2060 | 0.3251 | 0.4106 | 0 |
| **B26** | 0 | 0 | 0 | 0 | 0 | 0.1510 | 0 | 0 | 0 |
| **B28** | 0 | 0 | 0 | 0 | 0.1944 | 0 | 0 | 0 | 0.0471 |
| **H1** | 0 | 0 | 0 | 0 | 0 | 0 | 0 | 0 | 0 |
| **H3** | 0 | 0 | 0 | 0 | 0 | 0 | 0 | 0 | 0 |
| **H5** | 0 | 0 | 0 | 0 | 0 | 0 | 0 | 0 | 0 |
| **H7** | 0 | 0 | 0 | 0 | 0 | 0 | 0 | 0 | 0 |
| **H9** | 0 | 0 | 0 | 0 | 0 | 0 | 0 | 0 | 0 |
| **H11** | 0 | 0 | 0 | 0 | 0 | 0 | 0 | 0 | 0 |
| **H13** | 0 | 0 | 0 | 0 | 0 | 0 | 0 | 0 | 0 |
| **H2** | 0 | 0 | 0 | 0 | 0 | 0 | 0 | 0 | 0 |
| **H4** | 0 | 0 | -0.2334 | 0 | 0 | 0 | 0 | 0 | 0.1010 |
| **H6** | 0 | 0 | -0.5598 | 0 | 0 | 0 | 0 | 0 | 0 |
| **H8** | 0 | 0 | 0 | 0 | 0 | 0 | 0 | 0 | 0.1227 |
| **H10** | 0 | 0 | 0 | 0 | 0 | 0 | 0 | 0 | 0 |
| **H12** | 0 | 0 | 0 | 0 | 0 | 0 | 0 | 0 | 0 |
| **H14** | 0 | 0 | 0 | 0 | 0 | 0 | 0 | 0 | 0 |
| **AL** | 0 | 0 | 0 | 0 | 0 | 0 | 0 | 0 | 0 |
|  | **B2** | **B3** | **B4** | **B16** | **B17** | **B19** | **B21** | **B22** | **B23** |
| **SM** | 0 | 0 | 0 | 0 | 0 | 0 | 0 | 0 | 0 |
| **CA** | 0 | 0 | 0 | 0 | 0 | 0 | 0 | 0 | 0 |
| **XT** | 0 | 0 | 0 | 0 | 0 | 0 | 0 | 0 | 0 |
| **CO** | 0 | 0 | 0 | 0 | 0 | 0 | 0 | 0 | 0 |
| **OT** | 0 | 0 | 0 | 0 | 0 | 0 | 0 | 0 | 0 |
| **QM** | 0 | 0 | 0 | 0 | 0 | 0 | 0 | 0 | 0 |
| **QS** | 0 | 0 | 0 | 0 | 0 | 0 | 0 | 0 | 0 |
| **QA** | 0 | 0 | 0 | 0 | 0 | 0 | 0 | 0 | 0 |
| **QP** | 0 | 0 | 0 | 0 | 0 | 0 | 0 | 0 | 0 |
| **QV** | 0 | 0 | 0 | 0 | 0 | 0 | 0 | 0 | 0 |
| **T1** | 0 | 0 | 0 | 0 | 0 | 0 | 0 | 0 | 0 |
| **T2** | 0 | 0 | 0 | 0 | 0 | 0 | 0 | 0 | 0 |
| **T3** | 0 | 0 | 0 | 0 | 0 | 0 | 0 | 0 | 0 |
| **T4** | 0 | 0 | 0 | 0 | 0 | 0 | 0 | 0 | 0 |
| **T5** | 0 | 0 | 0 | 0 | 0 | 0 | 0 | 0 | 0 |
| **T6** | 0 | 0 | 0 | 0 | 0 | 0 | 0 | 0 | 0 |
| **T7** | 0 | 0 | 0 | 0 | 0 | 0 | 0 | 0 | 0 |
| **T8** | 0 | 0 | 0 | 0 | 0 | 0 | 0 | 0 | 0 |
| **PR** | 0 | 0 | 0 | 0 | 0 | 0 | 0 | 0 | 0 |
| **SO** | 0 | 0 | 0 | 0 | 0 | 0 | 0 | 0 | 0 |

|  | **B25** | **B30** | **B1** | **B7** | **B8** | **B10** | **B12** | **B13** | **B14** |
| --- | --- | --- | --- | --- | --- | --- | --- | --- | --- |
| **B3** | 0 | 0 | 0.5512 | 0.2738 | 0 | 0 | 0.4236 | 0 | 0.5079 |
| **B4** | 0 | 0 | 0 | 0 | 0 | 0 | 0 | 0 | 0 |
| **B16** | 0 | 0 | 0 | 0 | 0 | 0 | 0 | 0 | 0 |
| **B17** | 0 | 0 | 0 | 0 | 0 | 0 | 0 | 0.4247 | 0 |
| **B19** | 0 | 0 | 0.2026 | 0 | 0 | 0 | 0 | 0 | 0.7648 |
| **B21** | 0 | 0 | 0 | 0 | 0 | 0 | 0.2260 | 0 | 0.3529 |
| **B22** | 0 | 0 | 0 | 0 | 0 | 0 | 0 | 0 | 0 |
| **B23** | 0.3137 | 0 | 0 | 0 | 0 | 0.0911 | 0.0774 | 0 | 0 |
| **B25** | 0 | 0 | 0 | 0 | 0 | 0 | 0 | 0 | 0 |
| **B30** | 0 | 0 | 0 | 0 | 0 | 0.9194 | 0 | 0 | 0 |
| **B1** | 0 | 0 | 0.6774 | 0.5722 | 0 | 0.4819 | 0.4574 | 0.1483 | 0 |
| **B7** | 0 | 0.6774 | 0 | 1.1866 | 0 | 0.3007 | 0.3360 | 0 | 0 |
| **B8** | 0 | 0.5722 | 1.1866 | 0 | 0 | 0.1491 | 0.2307 | 0 | 0 |
| **B10** | 0 | 0 | 0 | 0 | 0 | 0.1228 | 0.9367 | 0.3765 | 0 |
| **B12** | 0.9194 | 0.4819 | 0.3007 | 0.1491 | 0.1228 | 0 | 0.2213 | 0.2796 | 0 |
| **B13** | 0 | 0.4574 | 0.3360 | 0.2307 | 0.9367 | 0.2213 | 0 | 0.0811 | 0.2979 |
| **B14** | 0 | 0.1483 | 0 | 0 | 0.3765 | 0.2796 | 0.0811 | 0 | 0 |
| **B15** | 0 | 0 | 0 | 0 | 0 | 0 | 0.2979 | 0 | 0 |
| **B18** | 0 | 0 | 0 | 0 | 0 | 0 | 0.8514 | 0 | 0 |
| **B27** | 0 | 0 | 0 | 0 | 0 | 0 | 0.1064 | 0 | 0 |
| **B29** | 0 | 0.6415 | 0 | 0 | 0 | 0 | 0 | 0 | 0 |
| **B5** | 0 | 0 | 0 | 0 | 0 | 0 | 0 | 0 | 0 |
| **B6** | 0 | 0.1388 | 0.0990 | 0 | 0.1048 | 0 | 0.1908 | 0 | 0 |
| **B9** | 0 | 0 | 0 | 0 | 0.1771 | 0 | 0 | 0 | 0 |
| **B11** | 0 | 0 | 0.4521 | 0 | 1.4954 | 0.2595 | 0.2545 | 0 | 0 |
|  | **B25** | **B30** | **B1** | **B7** | **B8** | **B10** | **B12** | **B13** | **B14** |
| **B20** | 0 | 0 | 0 | 0 | 0 | 0 | 0 | 0 | 0 |
| **B24** | 0 | 0 | 0 | 0.3582 | 0 | 0 | 0.6471 | 0 | 0 |
| **B26** | 0 | 0 | 0 | 0 | 0 | 0 | 0 | 0 | 0 |
| **B28** | 0 | 0 | 0 | 0 | 0 | 0 | 0 | 0 | 0 |
| **H1** | 0 | 0 | 0 | 0 | 0 | 0 | 0.1835 | 0 | 0 |
| **H3** | 0 | 0 | 0 | 0 | 0 | 0 | 0 | 0 | 0 |
| **H5** | 0.1408 | 0 | 0 | 0 | 0 | 0 | 0 | 0.3067 | 0 |
| **H7** | 0 | 0 | 0 | 0 | 0.1427 | 0 | 0 | 0 | 0 |
| **H9** | 0 | 0 | 0 | 0 | 0.1978 | 0 | 0 | 0 | 0 |
| **H11** | 0 | 0 | 0 | 0 | 0.1556 | 0 | 0 | 0 | 0 |
| **H13** | 0 | 0 | 0 | 0 | 0 | 0 | 0 | 0 | 0 |
| **H2** | 0 | 0 | 0 | 0 | 0.2561 | 0 | 0 | 0 | 0 |
| **H4** | 0 | 0 | 0 | 0 | 0 | 0 | 0 | 0 | 0 |
| **H6** | 0 | 0 | 0 | 0 | 0 | 0 | 0 | 0 | 0 |
| **H8** | 0 | 0 | 0 | 0 | 0.3236 | 0.1038 | 0.1718 | 0.0459 | 0 |
| **H10** | 0 | 0 | 0 | 0 | 0 | 0 | 0 | 0 | 0 |
| **H12** | 0 | 0 | 0 | 0 | 0.2073 | 0 | 0 | 0.0748 | 0 |
| **H14** | 0 | 0 | 0 | 0 | 0 | 0 | 0 | 0.1449 | 0 |
| **AL** | 0 | 0 | 0 | 0 | 0 | 0 | 0 | 0.2222 | 0 |
| **SM** | 0 | 0 | 0 | 0 | 0 | 0 | 0 | 0 | 0 |
| **CA** | 0.2077 | 0 | 0 | 0 | 0 | 0 | 0 | 0 | 0 |
| **XT** | 0 | 0 | 0 | 0 | 0 | 0 | 0 | 0 | 0 |
| **CO** | 0 | 0 | 0 | 0 | 0 | 0 | 0 | 0 | 0 |
| **OT** | 0 | 0 | 0 | 0 | 0 | 0 | 0 | 0 | 0 |
| **QM** | 0 | 0 | 0 | 0 | 0 | 0 | 0 | 0 | 0 |
| **QS** | 0 | 0 | 0 | 0 | 0 | 0 | 0 | 0 | 0 |
| **QA** | 0 | 0 | 0 | 0 | 0 | 0 | 0 | 0 | 0 |
| **QP** | 0 | 0 | 0 | 0 | 0 | 0 | 0 | 0 | 0 |
| **QV** | 0 | 0 | 0 | 0 | 0 | 0 | 0 | 0 | 0 |
| **T1** | 0 | 0 | 0 | 0 | 0 | 0 | 0 | 0 | 0 |
| **T2** | 0 | 0 | 0 | 0 | 0 | 0 | 0 | 0 | 0 |
| **T3** | 0 | 0 | 0 | 0 | 0 | 0 | 0 | 0 | 0 |
| **T4** | 0 | 0 | 0 | 0 | 0 | 0 | 0 | 0 | 0 |
| **T5** | 0 | 0 | 0 | 0 | 0 | 0 | 0 | 0 | 0 |
| **T6** | 0 | 0 | 0 | 0 | 0 | 0 | 0 | 0 | 0 |
| **T7** | 0 | 0 | 0 | 0 | 0 | 0 | 0 | 0 | 0 |
| **T8** | 0 | 0 | 0 | 0 | 0 | 0 | 0 | 0 | 0 |
| **PR** | 0 | 0 | 0 | 0 | 0 | 0 | 0 | 0 | 0 |
| **SO** | 0 | 0 | 0 | 0 | 0 | 0 | 0 | 0 | 0 |
| **B2** | 0 | 0 | 0 | 0 | 0 | 0 | 0 | 0 | 0 |

|  | **B15** | **B18** | **B27** | **B29** | **B5** | **B6** | **B9** | **B11** | **B20** |
| --- | --- | --- | --- | --- | --- | --- | --- | --- | --- |
| **B3** | 0 | 0 | 0 | 0 | 0.5215 | 0 | 0 | 0 | 0 |
| **B4** | 0 | 0 | 0 | 0 | 0 | 0 | 0 | 0 | 0 |
| **B16** | 0 | 0 | 0 | 0 | 0 | 0 | 0 | 0 | 0 |
| **B17** | 0 | 0 | 0 | 0 | 0 | 0 | 0 | 0 | 0 |
| **B19** | 0 | 0.2246 | 0 | 0 | 0.1368 | 0 | 0 | 0 | 0 |
|  | **B15** | **B18** | **B27** | **B29** | **B5** | **B6** | **B9** | **B11** | **B20** |
| **B21** | 0 | 0 | 0.0828 | 0 | 0 | 0 | 0 | 0 | 0 |
| **B22** | 0 | 0 | 0 | 0 | 0 | 0 | 0 | 0 | 0 |
| **B23** | 0 | 0.0463 | 0 | 0 | 0 | 0 | 0 | 0 | 0 |
| **B25** | 0 | 0.1960 | 0.1372 | 0 | 0.2050 | 0 | 0.1725 | 0 | 0 |
| **B30** | 0 | 0 | 0 | 0 | 0 | 0 | 0 | 0 | 0 |
| **B1** | 0 | 0 | 0.6415 | 0 | 0.1388 | 0 | 0 | 0 | 0 |
| **B7** | 0 | 0 | 0 | 0 | 0.0990 | 0 | 0.4521 | 0 | 0 |
| **B8** | 0 | 0 | 0 | 0 | 0 | 0 | 0 | 0 | 0.3582 |
| **B10** | 0 | 0 | 0 | 0 | 0.1048 | 0.1771 | 1.4954 | 0 | 0 |
| **B12** | 0 | 0 | 0 | 0 | 0 | 0 | 0.2595 | 0 | 0 |
| **B13** | 0.8514 | 0.1064 | 0 | 0 | 0.1908 | 0 | 0.2545 | 0 | 0.6471 |
| **B14** | 0 | 0 | 0 | 0 | 0 | 0 | 0 | 0 | 0 |
| **B15** | 0 | 0 | 0 | 0 | 0 | 0 | 0 | 0 | 0 |
| **B18** | 0 | 0 | 0 | 1.9274 | 0.1369 | 0 | 0 | 0 | 0 |
| **B27** | 0 | 0 | 0 | 0 | 0.1828 | 0.0872 | 0.4482 | 0.0863 | 0 |
| **B29** | 0 | 0 | 0 | 0 | 0 | 0 | 0 | 0 | 0 |
| **B5** | 1.9274 | 0 | 0 | 0 | 0 | 0 | 0 | 0 | 0 |
| **B6** | 0.1369 | 0.1828 | 0 | 0 | 0 | 0.4163 | 0.8505 | 0.2690 | 0 |
| **B9** | 0 | 0.0872 | 0 | 0 | 0.4163 | 0 | 0.1091 | 0 | 0 |
| **B11** | 0 | 0.4482 | 0 | 0 | 0.8505 | 0.1091 | 0 | 0 | 0 |
| **B20** | 0 | 0.0863 | 0 | 0 | 0.2690 | 0 | 0 | 0 | 0 |
| **B24** | 0 | 0 | 0 | 0 | 0 | 0 | 0 | 0 | 0 |
| **B26** | 0 | 0.0772 | 0 | 0 | 0 | 0 | 0.0397 | 0 | 0 |
| **B28** | 0 | 0 | 0 | 0 | 0.4372 | 0.6044 | 0.3928 | 0 | 0 |
| **H1** | 0 | 0.3947 | 0 | 0 | 0.1497 | 0.3876 | 0.3141 | 1.1332 | 0 |
| **H3** | 0 | 0 | 0 | 0 | 0 | 0 | 0 | 0 | 0 |
| **H5** | 0 | 0 | 0 | 0 | 0 | 0.1413 | 0 | 0.2089 | 0 |
| **H7** | 0 | 0 | 0 | 0 | 0 | 0 | 0.0521 | 0 | 0 |
| **H9** | 0 | 0 | 0 | 0 | 0 | 0.3018 | 0.2406 | 0 | 0 |
| **H11** | 0 | 0 | 0 | 0 | 0 | 0.0783 | 0 | 0 | 0 |
| **H13** | 0 | 0 | 0 | 0 | 0 | 0.5154 | 0.1770 | 0 | 0 |
| **H2** | 0 | 0 | 0 | 0 | 0 | 0.3152 | 0 | 0 | 0 |
| **H4** | 0 | 0 | 0 | 0 | 0 | 0 | 0 | 0 | 0 |
| **H6** | 0 | 0 | 0 | 0 | 0 | 0 | 0.2764 | 0 | 0 |
| **H8** | 0 | 0 | 0 | 0 | 0 | 0.1744 | 0 | 0 | 0 |
| **H10** | 0 | 0 | 0 | 0 | 0 | 0 | 0.0678 | 0 | 0 |
| **H12** | 0 | 0 | 0 | 0 | 0 | 0 | 0 | 0 | 0 |
| **H14** | 0 | 0 | 0 | 0 | 0.0948 | 0 | 0 | 0 | 0 |
| **AL** | 0 | 0.0914 | 0 | 0 | 0 | 0 | 0 | 0.2445 | 0 |
| **SM** | 0 | 0 | 0 | 0 | 0 | 0 | 0 | 0 | 0 |
| **CA** | 0 | 0 | 0 | 0 | 0 | 0 | 0 | 0 | 0 |
| **XT** | 0 | 0 | 0 | 0 | 0 | 0 | 0 | 0 | 0 |
| **CO** | 0 | 0 | 0 | 0 | 0 | 0 | 0 | 0 | 0 |
| **OT** | 0 | 0 | 0 | 0 | 0 | 0 | 0 | 0 | 0 |
| **QM** | 0 | 0 | 0 | 0 | 0 | 0 | 0 | 0 | 0 |
| **QS** | 0 | 0 | 0 | 0 | 0 | 0 | 0 | 0 | 0 |
| **QA** | 0 | 0 | 0 | 0 | 0 | 0 | 0 | 0 | 0 |
|  | **B15** | **B18** | **B27** | **B29** | **B5** | **B6** | **B9** | **B11** | **B20** |
| **QP** | 0 | 0 | 0 | 0 | 0 | 0 | 0.1533 | 0 | 0 |
| **QV** | 0 | 0 | 0 | 0 | 0 | 0 | 0 | 0 | 0 |
| **T1** | 0 | 0 | 0 | 0 | 0 | 0 | 0 | 0 | 0 |
| **T2** | 0 | 0 | 0 | 0 | 0 | 0 | 0 | 0 | 0 |
| **T3** | 0 | 0 | 0 | 0 | 0 | 0 | 0 | 0 | 0 |
| **T4** | 0 | 0 | 0 | 0 | 0 | 0 | 0 | 0 | 0 |
| **T5** | 0 | 0 | 0 | 0 | 0 | 0 | 0 | 0 | 0 |
| **T6** | 0 | 0 | 0 | 0 | 0 | 0 | 0 | 0 | 0 |
| **T7** | 0 | 0 | 0 | 0 | 0 | 0 | 0 | 0 | 0 |
| **T8** | 0 | 0 | 0 | 0 | 0 | 0 | 0 | 0 | 0 |
| **PR** | 0 | 0 | 0 | 0 | 0 | 0 | 0.1354 | 0 | 0 |
| **SO** | 0 | 0 | 0 | 0 | 0 | 0 | 0 | 0 | 0 |

|  | **B24** | | **B26** | **B28** | **H1** | **H3** | **H5** | **H7** | **H9** | **H11** |
| --- | --- | --- | --- | --- | --- | --- | --- | --- | --- | --- |
| **B2** | | 0 | 0 | 0 | 0 | 0 | 0 | 0 | 0 | 0 |
| **B3** | | 0 | 0 | 0 | 0 | 0 | 0 | 0 | 0 | 0 |
| **B4** | | 0 | 0 | 0 | 0 | 0 | 0 | 0 | 0 | 0 |
| **B16** | | 0.4176 | 0 | 0 | 0 | 0 | 0 | 0 | 0 | 0 |
| **B17** | | 0 | 0 | 0.1944 | 0 | 0 | 0 | 0 | 0 | 0 |
| **B19** | | 0.2060 | 0.1510 | 0 | 0 | 0 | 0 | 0 | 0 | 0 |
| **B21** | | 0.3251 | 0 | 0 | 0 | 0 | 0 | 0 | 0 | 0 |
| **B22** | | 0.4106 | 0 | 0 | 0 | 0 | 0 | 0 | 0 | 0 |
| **B23** | | 0 | 0 | 0.0471 | 0 | 0 | 0 | 0 | 0 | 0 |
| **B25** | | 0 | 0 | 0 | 0 | 0.1408 | 0 | 0 | 0 | 0 |
| **B30** | | 0 | 0 | 0 | 0 | 0 | 0 | 0 | 0 | 0 |
| **B1** | | 0 | 0 | 0 | 0 | 0 | 0 | 0 | 0 | 0 |
| **B7** | | 0 | 0 | 0 | 0 | 0 | 0 | 0 | 0 | 0 |
| **B8** | | 0 | 0 | 0 | 0 | 0 | 0.1427 | 0.1978 | 0.1556 | 0 |
| **B10** | | 0 | 0 | 0 | 0 | 0 | 0 | 0 | 0 | 0 |
| **B12** | | 0 | 0 | 0.1835 | 0 | 0 | 0 | 0 | 0 | 0 |
| **B13** | | 0 | 0 | 0 | 0 | 0.3067 | 0 | 0 | 0 | 0 |
| **B14** | | 0 | 0 | 0 | 0 | 0 | 0 | 0 | 0 | 0 |
| **B15** | | 0 | 0 | 0 | 0 | 0 | 0 | 0 | 0 | 0 |
| **B18** | | 0.0772 | 0 | 0.3947 | 0 | 0 | 0 | 0 | 0 | 0 |
| **B27** | | 0 | 0 | 0 | 0 | 0 | 0 | 0 | 0 | 0 |
| **B29** | | 0 | 0 | 0 | 0 | 0 | 0 | 0 | 0 | 0 |
| **B5** | | 0 | 0.4372 | 0.1497 | 0 | 0 | 0 | 0 | 0 | 0 |
| **B6** | | 0 | 0.6044 | 0.3876 | 0 | 0.1413 | 0 | 0.3018 | 0.0783 | 0.5154 |
| **B9** | | 0.0397 | 0.3928 | 0.3141 | 0 | 0 | 0.0521 | 0.2406 | 0 | 0.1770 |
| **B11** | | 0 | 0 | 1.1332 | 0 | 0.2089 | 0 | 0 | 0 | 0 |
| **B20** | | 0 | 0 | 0 | 0 | 0 | 0 | 0 | 0 | 0 |
| **B24** | | 0 | 0 | 0 | 0 | 0 | 0 | 0 | 0 | 0 |
| **B26** | | 0 | 0 | 0.2974 | 0.1854 | 0 | 0 | 0 | 0 | 0 |
| **B28** | | 0 | 0.2974 | 0 | 0 | 0 | 0 | 0.1852 | 0 | 0.3626 |
| **H1** | | 0 | 0.1854 | 0 | 0 | 0.5146 | 0.6345 | 0.6944 | 0 | 0.8841 |
| **H3** | | 0 | 0 | 0 | 0.5146 | 0 | 0.4705 | 0 | 0.7097 | 0.2415 |
| **H5** | | 0 | 0 | 0 | 0.6345 | 0.4705 | 0 | 0 | 0 | 0.1513 |
|  | | **B24** | **B26** | **B28** | **H1** | **H3** | **H5** | **H7** | **H9** | **H11** |
| **H7** | | 0 | 0 | 0.1852 | 0.6944 | 0 | 0 | 0 | 0.0716 | 0.8769 |
| **H9** | | 0 | 0 | 0 | 0 | 0.7097 | 0 | 0.0716 | 0 | 0.5358 |
| **H11** | | 0 | 0 | 0.3626 | 0.8841 | 0.2415 | 0.1513 | 0.8769 | 0.5358 | 0 |
| **H13** | | 0 | 0 | 0 | 0.1571 | 1.3434 | 0.5050 | 0.1780 | 0.3986 | 1.0531 |
| **H2** | | 0 | 0 | 0 | 0 | 0 | 0.1263 | 0 | 0 | 0 |
| **H4** | | 0 | 0 | 0 | 0 | 0 | 0.1721 | 0.0958 | 0 | 0.2008 |
| **H6** | | 0 | 0 | 0 | 0 | 0.0632 | 0.6160 | 0.0630 | 0 | 0 |
| **H8** | | 0 | 0 | 0 | 0.3246 | 0.4824 | 0.1039 | 0.1640 | 0.1728 | 0.7104 |
| **H10** | | 0 | 0 | 0 | 0 | 0 | 0.0868 | 0 | 0.1398 | 0 |
| **H12** | | 0 | 0 | 0 | 0 | 0.2779 | 0 | 0.1519 | 0 | 0.0602 |
| **H14** | | 0 | 0 | 0 | 0 | 0 | 0 | 0.3948 | 0.1729 | 0 |
| **AL** | | 0 | 0 | 0 | 0 | 0 | 0 | 0 | 0 | 0 |
| **SM** | | 0 | 0 | 0 | 0 | 0 | 0 | 0 | 0 | 0 |
| **CA** | | 0 | 0 | 0 | 0 | 0 | 0 | 0 | 0 | 0 |
| **XT** | | 0 | 0 | 0 | 0 | 0 | 0 | 0 | 0 | 0 |
| **CO** | | 0 | 0 | 0 | 0 | 0 | 0 | 0 | 0 | 0 |
| **OT** | | 0 | 0 | 0 | 0 | 0 | 0 | 0 | 0 | 0 |
| **QM** | | 0 | 0 | 0 | 0 | 0 | 0 | 0 | 0 | 0 |
| **QS** | | 0 | 0 | 0 | 0 | 0 | 0 | 0 | 0 | 0 |
| **QA** | | 0 | 0 | 0 | 0 | 0 | 0 | 0 | 0 | 0 |
| **QP** | | 0 | 0 | 0 | 0 | 0 | 0 | 0.0410 | 0 | 0 |
| **QV** | | 0 | 0 | 0 | 0 | 0 | 0 | 0 | -0.0973 | 0 |
| **T1** | | 0 | 0 | 0 | 0 | 0 | 0 | 0 | 0 | 0 |
| **T2** | | 0 | 0 | 0 | 0 | 0 | 0 | 0 | 0 | 0 |
| **T3** | | 0 | 0 | 0 | 0 | 0 | 0 | 0 | 0 | 0 |
| **T4** | | 0 | 0 | 0 | 0 | 0 | 0 | 0 | 0 | 0 |
| **T5** | | 0 | 0 | 0 | 0 | 0 | 0 | 0 | 0 | 0 |
| **T6** | | 0 | 0 | 0 | 0 | 0 | 0 | 0 | 0 | 0 |
| **T7** | | 0 | 0 | 0 | 0 | 0 | 0 | 0 | 0 | 0 |
| **T8** | | 0 | 0 | 0 | 0 | 0 | 0 | 0 | 0 | 0 |
| **PR** | | 0 | 0 | 0 | 0 | 0 | 0 | 0 | 0 | 0 |
| **SO** | | 0 | 0 | 0 | 0 | 0 | 0 | 0 | 0 | 0 |

|  | **H13** | **H2** | **H4** | **H6** | **H8** | **H10** | **H12** | **H14** | **AL** |
| --- | --- | --- | --- | --- | --- | --- | --- | --- | --- |
| **B2** | 0 | 0 | 0 | 0 | 0 | 0 | 0 | 0 | 0 |
| **B3** | 0 | 0 | 0 | 0 | 0 | 0 | 0 | 0 | 0 |
| **B4** | 0 | 0 | -0.2334 | -0.5598 | 0 | 0 | 0 | 0 | 0 |
| **B16** | 0 | 0 | 0 | 0 | 0 | 0 | 0 | 0 | 0 |
| **B17** | 0 | 0 | 0 | 0 | 0 | 0 | 0 | 0 | 0 |
| **B19** | 0 | 0 | 0 | 0 | 0 | 0 | 0 | 0 | 0 |
| **B21** | 0 | 0 | 0 | 0 | 0 | 0 | 0 | 0 | 0 |
| **B22** | 0 | 0 | 0 | 0 | 0 | 0 | 0 | 0 | 0 |
| **B23** | 0 | 0 | 0.1010 | 0 | 0.1227 | 0 | 0 | 0 | 0 |
| **B25** | 0 | 0 | 0 | 0 | 0 | 0 | 0 | 0 | 0 |
| **B30** | 0 | 0 | 0 | 0 | 0 | 0 | 0 | 0 | 0 |
| **B1** | 0 | 0 | 0 | 0 | 0 | 0 | 0 | 0 | 0 |
| **B7** | 0 | 0 | 0 | 0 | 0 | 0 | 0 | 0 | 0 |
|  | **H13** | **H2** | **H4** | **H6** | **H8** | **H10** | **H12** | **H14** | **AL** |
| **B8** | 0.2561 | 0 | 0 | 0.3236 | 0 | 0.2073 | 0 | 0 | 0 |
| **B10** | 0 | 0 | 0 | 0.1038 | 0 | 0 | 0 | 0 | 0 |
| **B12** | 0 | 0 | 0 | 0.1718 | 0 | 0 | 0 | 0 | 0 |
| **B13** | 0 | 0 | 0 | 0.0459 | 0 | 0.0748 | 0.1449 | 0.2222 | 0 |
| **B14** | 0 | 0 | 0 | 0 | 0 | 0 | 0 | 0 | 0 |
| **B15** | 0 | 0 | 0 | 0 | 0 | 0 | 0 | 0 | 0 |
| **B18** | 0 | 0 | 0 | 0 | 0 | 0 | 0 | 0.0914 | 0 |
| **B27** | 0 | 0 | 0 | 0 | 0 | 0 | 0 | 0 | 0 |
| **B29** | 0 | 0 | 0 | 0 | 0 | 0 | 0 | 0 | 0 |
| **B5** | 0 | 0 | 0 | 0 | 0 | 0 | 0.0948 | 0 | 0 |
| **B6** | 0.3152 | 0 | 0 | 0.1744 | 0 | 0 | 0 | 0 | 0 |
| **B9** | 0 | 0 | 0.2764 | 0 | 0.0678 | 0 | 0 | 0 | 0 |
| **B11** | 0 | 0 | 0 | 0 | 0 | 0 | 0 | 0.2445 | 0 |
| **B20** | 0 | 0 | 0 | 0 | 0 | 0 | 0 | 0 | 0 |
| **B24** | 0 | 0 | 0 | 0 | 0 | 0 | 0 | 0 | 0 |
| **B26** | 0 | 0 | 0 | 0 | 0 | 0 | 0 | 0 | 0 |
| **B28** | 0 | 0 | 0 | 0 | 0 | 0 | 0 | 0 | 0 |
| **H1** | 0.1571 | 0 | 0 | 0 | 0.3246 | 0 | 0 | 0 | 0 |
| **H3** | 1.3434 | 0 | 0 | 0.0632 | 0.4824 | 0 | 0.2779 | 0 | 0 |
| **H5** | 0.5050 | 0.1263 | 0.1721 | 0.6160 | 0.1039 | 0.0868 | 0 | 0 | 0 |
| **H7** | 0.1780 | 0 | 0.0958 | 0.0630 | 0.1640 | 0 | 0.1519 | 0.3948 | 0 |
| **H9** | 0.3986 | 0 | 0 | 0 | 0.1728 | 0.1398 | 0 | 0.1729 | 0 |
| **H11** | 1.0531 | 0 | 0.2008 | 0 | 0.7104 | 0 | 0.0602 | 0 | 0 |
| **H13** | 0 | 0.0814 | 0.2137 | 0.3087 | 0 | 0 | 0 | 0 | 0 |
| **H2** | 0.0814 | 0 | 1.1180 | 0.2939 | 0.7565 | 0 | 1.2091 | 0 | 0 |
| **H4** | 0.2137 | 1.1180 | 0 | 0.7677 | 0.2268 | 0 | 1.0821 | 0.4082 | 0 |
| **H6** | 0.3087 | 0.2939 | 0.7677 | 0 | 0.4018 | 0.3613 | 0.4396 | 0.5937 | 0 |
| **H8** | 0 | 0.7565 | 0.2268 | 0.4018 | 0 | 0.2798 | 0.2032 | 0 | 0 |
| **H10** | 0 | 0 | 0 | 0.3613 | 0.2798 | 0 | 0.4695 | 0.0569 | 0 |
| **H12** | 0 | 1.2091 | 1.0821 | 0.4396 | 0.2032 | 0.4695 | 0 | 0.2429 | 0 |
| **H14** | 0 | 0 | 0.4082 | 0.5937 | 0 | 0.0569 | 0.2429 | 0 | 0 |
| **AL** | 0 | 0 | 0 | 0 | 0 | 0 | 0 | 0 | 0 |
| **SM** | 0 | 0 | 0 | 0 | 0 | 0 | 0 | 0 | 0 |
| **CA** | 0 | 0 | 0 | 0 | 0 | 0 | 0 | 0 | 0 |
| **XT** | 0 | 0 | 0 | 0 | 0 | 0 | 0 | 0 | 0 |
| **CO** | 0 | 0 | 0 | 0 | 0 | 0 | 0 | 0 | 0 |
| **OT** | 0 | 0 | 0 | 0 | 0 | 0 | 0 | 0 | 0 |
| **QM** | 0 | 0 | 0 | 0 | 0 | 0 | 0 | 0 | 0 |
| **QS** | 0 | 0 | 0 | 0 | 0 | 0 | 0 | 0 | 0 |
| **QA** | 0.1356 | 0 | 0 | 0 | 0.6030 | 0 | 0.1473 | 0 | 0 |
| **QP** | 0 | 0 | 0 | 0 | 0.7473 | 0 | 0 | 0 | 0 |
| **QV** | 0 | -0.1651 | 0 | -0.3343 | -0.3510 | -0.0994 | 0 | 0 | 0 |
| **T1** | 0 | 0 | 0 | 0 | 0 | 0 | 0 | 0 | 0 |
| **T2** | 0 | 0 | 0 | 0 | 0 | 0 | 0 | 0 | 0 |
| **T3** | 0 | 0 | 0 | 0 | 0 | 0 | 0 | 0 | 0 |
| **T4** | 0 | 0 | 0 | 0 | 0 | 0 | 0 | 0 | 0 |
| **T5** | 0 | 0 | 0 | 0 | 0 | 0 | 0 | 0 | 0 |
|  | **H13** | **H2** | **H4** | **H6** | **H8** | **H10** | **H12** | **H14** | **AL** |
| **T6** | 0 | 0 | 0 | 0 | 0 | 0 | 0 | 0 | 0 |
| **T7** | 0 | 0 | 0 | 0 | 0 | 0 | 0 | 0 | 0 |
| **T8** | 0 | 0 | 0 | 0 | 0 | 0 | 0 | 0 | 0 |
| **PR** | 0 | 0 | 0 | 0 | 0 | 0 | 0 | 0 | 0 |
| **SO** | 0 | 0 | 0 | 0 | 0 | 0 | 0 | 0 | 0 |

|  | **SM** | **CA** | **XT** | **CO** | **OT** | **QM** | **QS** | **QA** | **QP** |
| --- | --- | --- | --- | --- | --- | --- | --- | --- | --- |
| **B2** | 0 | 0 | 0 | 0 | 0 | 0 | 0 | 0 | 0 |
| **B3** | 0 | 0 | 0 | 0 | 0 | 0 | 0 | 0 | 0 |
| **B4** | 0 | 0 | 0 | 0 | 0 | 0 | 0 | 0 | 0 |
| **B16** | 0 | 0 | 0 | 0 | 0 | 0 | 0 | 0 | 0 |
| **B17** | 0 | 0 | 0 | 0 | 0 | 0 | 0 | 0 | 0 |
| **B19** | 0 | 0 | 0 | 0 | 0 | 0 | 0 | 0 | 0 |
| **B21** | 0 | 0 | 0 | 0 | 0 | 0 | 0 | 0 | 0 |
| **B22** | 0 | 0 | 0 | 0 | 0 | 0 | 0 | 0 | 0 |
| **B23** | 0 | 0 | 0 | 0 | 0 | 0 | 0 | 0 | 0 |
| **B25** | 0.2077 | 0 | 0 | 0 | 0 | 0 | 0 | 0 | 0 |
| **B30** | 0 | 0 | 0 | 0 | 0 | 0 | 0 | 0 | 0 |
| **B1** | 0 | 0 | 0 | 0 | 0 | 0 | 0 | 0 | 0 |
| **B7** | 0 | 0 | 0 | 0 | 0 | 0 | 0 | 0 | 0 |
| **B8** | 0 | 0 | 0 | 0 | 0 | 0 | 0 | 0 | 0 |
| **B10** | 0 | 0 | 0 | 0 | 0 | 0 | 0 | 0 | 0 |
| **B12** | 0 | 0 | 0 | 0 | 0 | 0 | 0 | 0 | 0 |
| **B13** | 0 | 0 | 0 | 0 | 0 | 0 | 0 | 0 | 0 |
| **B14** | 0 | 0 | 0 | 0 | 0 | 0 | 0 | 0 | 0 |
| **B15** | 0 | 0 | 0 | 0 | 0 | 0 | 0 | 0 | 0 |
| **B18** | 0 | 0 | 0 | 0 | 0 | 0 | 0 | 0 | 0 |
| **B27** | 0 | 0 | 0 | 0 | 0 | 0 | 0 | 0 | 0 |
| **B29** | 0 | 0 | 0 | 0 | 0 | 0 | 0 | 0 | 0 |
| **B5** | 0 | 0 | 0 | 0 | 0 | 0 | 0 | 0 | 0 |
| **B6** | 0 | 0 | 0 | 0 | 0 | 0 | 0 | 0 | 0 |
| **B9** | 0 | 0 | 0 | 0 | 0 | 0 | 0 | 0 | 0.1533 |
| **B11** | 0 | 0 | 0 | 0 | 0 | 0 | 0 | 0 | 0 |
| **B20** | 0 | 0 | 0 | 0 | 0 | 0 | 0 | 0 | 0 |
| **B24** | 0 | 0 | 0 | 0 | 0 | 0 | 0 | 0 | 0 |
| **B26** | 0 | 0 | 0 | 0 | 0 | 0 | 0 | 0 | 0 |
| **B28** | 0 | 0 | 0 | 0 | 0 | 0 | 0 | 0 | 0 |
| **H1** | 0 | 0 | 0 | 0 | 0 | 0 | 0 | 0 | 0 |
| **H3** | 0 | 0 | 0 | 0 | 0 | 0 | 0 | 0 | 0 |
| **H5** | 0 | 0 | 0 | 0 | 0 | 0 | 0 | 0 | 0 |
| **H7** | 0 | 0 | 0 | 0 | 0 | 0 | 0 | 0 | 0.0410 |
| **H9** | 0 | 0 | 0 | 0 | 0 | 0 | 0 | 0 | 0 |
| **H11** | 0 | 0 | 0 | 0 | 0 | 0 | 0 | 0 | 0 |
| **H13** | 0 | 0 | 0 | 0 | 0 | 0 | 0 | 0.1356 | 0 |
| **H2** | 0 | 0 | 0 | 0 | 0 | 0 | 0 | 0 | 0 |
| **H4** | 0 | 0 | 0 | 0 | 0 | 0 | 0 | 0 | 0 |
| **H6** | 0 | 0 | 0 | 0 | 0 | 0 | 0 | 0 | 0 |
|  | **SM** | **CA** | **XT** | **CO** | **OT** | **QM** | **QS** | **QA** | **QP** |
| **H8** | 0 | 0 | 0 | 0 | 0 | 0 | 0 | 0.6030 | 0.7473 |
| **H10** | 0 | 0 | 0 | 0 | 0 | 0 | 0 | 0 | 0 |
| **H12** | 0 | 0 | 0 | 0 | 0 | 0 | 0 | 0.1473 | 0 |
| **H14** | 0 | 0 | 0 | 0 | 0 | 0 | 0 | 0 | 0 |
| **AL** | 0 | 0 | 0 | 0 | 0 | 0 | 0 | 0 | 0 |
| **SM** | 0 | 1.1123 | 0 | 0.2795 | 0 | 0 | 0 | 0 | 0 |
| **CA** | 1.1123 | 0 | 0.9440 | 0 | 0.4830 | 0 | 0 | 0 | 0 |
| **XT** | 0 | 0.9440 | 0 | 0.5653 | 1.3545 | 0 | 0 | 0 | 0 |
| **CO** | 0.2795 | 0 | 0.5653 | 0 | 0.7342 | 0 | 0 | 0 | 0 |
| **OT** | 0 | 0.4830 | 1.3545 | 0.7342 | 0 | 0 | 0 | 0 | 0 |
| **QM** | 0 | 0 | 0 | 0 | 0 | 0 | 1.1549 | 1.3815 | 1.3347 |
| **QS** | 0 | 0 | 0 | 0 | 0 | 1.1549 | 0 | 1.5410 | 0 |
| **QA** | 0 | 0 | 0 | 0 | 0 | 1.3815 | 1.5410 | 0 | 0.3111 |
| **QP** | 0 | 0 | 0 | 0 | 0 | 1.3347 | 0 | 0.3111 | 0 |
| **QV** | 0 | 0 | 0 | 0 | 0 | -0.2254 | 0 | -0.6810 | -0.4406 |
| **T1** | 0 | 0 | 0 | 0 | 0 | 0 | 0 | 0 | 0 |
| **T2** | 0 | 0 | 0 | 0 | 0 | 0 | 0 | 0 | 0 |
| **T3** | 0 | 0 | 0 | 0 | 0 | 0 | 0 | 0 | 0 |
| **T4** | 0 | 0 | 0 | 0 | 0 | 0 | 0 | 0 | 0 |
| **T5** | 0 | 0 | 0 | 0 | 0 | 0 | 0 | 0 | 0 |
| **T6** | 0 | 0 | 0 | 0 | 0 | 0 | 0 | 0 | 0 |
| **T7** | 0 | 0 | 0 | 0 | 0 | 0 | 0 | 0 | 0 |
| **T8** | 0 | 0 | 0 | 0 | 0 | 0 | 0 | 0 | 0 |
| **PR** | 0 | 0 | 1.0892 | 0 | 1.1404 | -0.1247 | 0 | 0 | 0 |
| **SO** | 0 | 0 | 0.3289 | 0 | 0 | 0 | 0 | 0 | 0 |

|  | **QV** | **T1** | **T2** | **T3** | **T4** | **T5** | **T6** | **T7** | **T8** |
| --- | --- | --- | --- | --- | --- | --- | --- | --- | --- |
| **B2** | 0 | 0 | 0 | 0 | 0 | 0 | 0 | 0 | 0 |
| **B3** | 0 | 0 | 0 | 0 | 0 | 0 | 0 | 0 | 0 |
| **B4** | 0 | 0 | 0 | 0 | 0 | 0 | 0 | 0 | 0 |
| **B16** | 0 | 0 | 0 | 0 | 0 | 0 | 0 | 0 | 0 |
| **B17** | 0 | 0 | 0 | 0 | 0 | 0 | 0 | 0 | 0 |
| **B19** | 0 | 0 | 0 | 0 | 0 | 0 | 0 | 0 | 0 |
| **B21** | 0 | 0 | 0 | 0 | 0 | 0 | 0 | 0 | 0 |
| **B22** | 0 | 0 | 0 | 0 | 0 | 0 | 0 | 0 | 0 |
| **B23** | 0 | 0 | 0 | 0 | 0 | 0 | 0 | 0 | 0 |
| **B25** | 0 | 0 | 0 | 0 | 0 | 0 | 0 | 0 | 0 |
| **B30** | 0 | 0 | 0 | 0 | 0 | 0 | 0 | 0 | 0 |
| **B1** | 0 | 0 | 0 | 0 | 0 | 0 | 0 | 0 | 0 |
| **B7** | 0 | 0 | 0 | 0 | 0 | 0 | 0 | 0 | 0 |
| **B8** | 0 | 0 | 0 | 0 | 0 | 0 | 0 | 0 | 0 |
| **B10** | 0 | 0 | 0 | 0 | 0 | 0 | 0 | 0 | 0 |
| **B12** | 0 | 0 | 0 | 0 | 0 | 0 | 0 | 0 | 0 |
| **B13** | 0 | 0 | 0 | 0 | 0 | 0 | 0 | 0 | 0 |
| **B14** | 0 | 0 | 0 | 0 | 0 | 0 | 0 | 0 | 0 |
| **B15** | 0 | 0 | 0 | 0 | 0 | 0 | 0 | 0 | 0 |
| **B18** | 0 | 0 | 0 | 0 | 0 | 0 | 0 | 0 | 0 |
|  | **QV** | **T1** | **T2** | **T3** | **T4** | **T5** | **T6** | **T7** | **T8** |
| **B27** | 0 | 0 | 0 | 0 | 0 | 0 | 0 | 0 | 0 |
| **B29** | 0 | 0 | 0 | 0 | 0 | 0 | 0 | 0 | 0 |
| **B5** | 0 | 0 | 0 | 0 | 0 | 0 | 0 | 0 | 0 |
| **B6** | 0 | 0 | 0 | 0 | 0 | 0 | 0 | 0 | 0 |
| **B9** | 0 | 0 | 0 | 0 | 0 | 0 | 0 | 0 | 0 |
| **B11** | 0 | 0 | 0 | 0 | 0 | 0 | 0 | 0 | 0 |
| **B20** | 0 | 0 | 0 | 0 | 0 | 0 | 0 | 0 | 0 |
| **B24** | 0 | 0 | 0 | 0 | 0 | 0 | 0 | 0 | 0 |
| **B26** | 0 | 0 | 0 | 0 | 0 | 0 | 0 | 0 | 0 |
| **B28** | 0 | 0 | 0 | 0 | 0 | 0 | 0 | 0 | 0 |
| **H1** | 0 | 0 | 0 | 0 | 0 | 0 | 0 | 0 | 0 |
| **H3** | 0 | 0 | 0 | 0 | 0 | 0 | 0 | 0 | 0 |
| **H5** | 0 | 0 | 0 | 0 | 0 | 0 | 0 | 0 | 0 |
| **H7** | 0 | 0 | 0 | 0 | 0 | 0 | 0 | 0 | 0 |
| **H9** | -0.0973 | 0 | 0 | 0 | 0 | 0 | 0 | 0 | 0 |
| **H11** | 0 | 0 | 0 | 0 | 0 | 0 | 0 | 0 | 0 |
| **H13** | 0 | 0 | 0 | 0 | 0 | 0 | 0 | 0 | 0 |
| **H2** | -0.1651 | 0 | 0 | 0 | 0 | 0 | 0 | 0 | 0 |
| **H4** | 0 | 0 | 0 | 0 | 0 | 0 | 0 | 0 | 0 |
| **H6** | -0.3343 | 0 | 0 | 0 | 0 | 0 | 0 | 0 | 0 |
| **H8** | -0.3510 | 0 | 0 | 0 | 0 | 0 | 0 | 0 | 0 |
| **H10** | -0.0994 | 0 | 0 | 0 | 0 | 0 | 0 | 0 | 0 |
| **H12** | 0 | 0 | 0 | 0 | 0 | 0 | 0 | 0 | 0 |
| **H14** | 0 | 0 | 0 | 0 | 0 | 0 | 0 | 0 | 0 |
| **AL** | 0 | 0 | 0 | 0 | 0 | 0 | 0 | 0 | 0 |
| **SM** | 0 | 0 | 0 | 0 | 0 | 0 | 0 | 0 | 0 |
| **CA** | 0 | 0 | 0 | 0 | 0 | 0 | 0 | 0 | 0 |
| **XT** | 0 | 0 | 0 | 0 | 0 | 0 | 0 | 0 | 0 |
| **CO** | 0 | 0 | 0 | 0 | 0 | 0 | 0 | 0 | 0 |
| **OT** | 0 | 0 | 0 | 0 | 0 | 0 | 0 | 0 | 0 |
| **QM** | -0.2254 | 0 | 0 | 0 | 0 | 0 | 0 | 0 | 0 |
| **QS** | 0 | 0 | 0 | 0 | 0 | 0 | 0 | 0 | 0 |
| **QA** | -0.6810 | 0 | 0 | 0 | 0 | 0 | 0 | 0 | 0 |
| **QP** | -0.4406 | 0 | 0 | 0 | 0 | 0 | 0 | 0 | 0 |
| **QV** | 0 | 0 | 0 | 0 | 0 | 0 | 0 | 0 | 0 |
| **T1** | 0 | 0 | 2.2072 | 0 | 0.6891 | 0 | 0 | 0 | 2.8340 |
| **T2** | 0 | 2.2072 | 0 | 0 | 0.5660 | 0.4246 | 0 | 0.2935 | 0 |
| **T3** | 0 | 0 | 0 | 0 | 0 | 0 | 0 | 0 | 0 |
| **T4** | 0 | 0.6891 | 0.5660 | 0 | 0 | 0 | 0 | 0 | 0 |
| **T5** | 0 | 0 | 0.4246 | 0 | 0 | 0 | 0 | 0.5820 | 0 |
| **T6** | 0 | 0 | 0 | 0 | 0 | 0 | 0 | 0 | 0 |
| **T7** | 0 | 0 | 0.2935 | 0 | 0 | 0.5820 | 0 | 0 | 0.2957 |
| **T8** | 0 | 2.8340 | 0 | 0 | 0 | 0 | 0 | 0.2957 | 0 |
| **PR** | 0 | 0 | 0 | 0 | 0 | 0 | 0 | 0 | 0 |
| **SO** | 0 | 0 | 0 | 0 | 0 | 0 | 0 | 0 | 0 |

|  | **PR** | **SO** |
| --- | --- | --- |
| **B2** | 0 | 0 |
| **B3** | 0 | 0 |
| **B4** | 0 | 0 |
| **B16** | 0 | 0 |
| **B17** | 0 | 0 |
| **B19** | 0 | 0 |
| **B21** | 0 | 0 |
| **B22** | 0 | 0 |
| **B23** | 0 | 0 |
| **B25** | 0 | 0 |
| **B30** | 0 | 0 |
| **B1** | 0 | 0 |
| **B7** | 0 | 0 |
| **B8** | 0 | 0 |
| **B10** | 0 | 0 |
| **B12** | 0 | 0 |
| **B13** | 0 | 0 |
| **B14** | 0 | 0 |
| **B15** | 0 | 0 |
| **B18** | 0 | 0 |
| **B27** | 0 | 0 |
| **B29** | 0 | 0 |
| **B5** | 0 | 0 |
| **B6** | 0 | 0 |
| **B9** | 0.1354 | 0 |
| **B11** | 0 | 0 |
| **B20** | 0 | 0 |
| **B24** | 0 | 0 |
| **B26** | 0 | 0 |
| **B28** | 0 | 0 |
| **H1** | 0 | 0 |
| **H3** | 0 | 0 |
| **H5** | 0 | 0 |
| **H7** | 0 | 0 |
| **H9** | 0 | 0 |
| **H11** | 0 | 0 |
| **H13** | 0 | 0 |
| **H2** | 0 | 0 |
| **H4** | 0 | 0 |
| **H6** | 0 | 0 |
| **H8** | 0 | 0 |
| **H10** | 0 | 0 |
| **H12** | 0 | 0 |
| **H14** | 0 | 0 |
| **AL** | 0 | 0 |
| **SM** | 0 | 0 |
| **CA** | 0 | 0 |
|  | **PR** | **SO** |
| **XT** | 1.0892 | 0.3289 |
| **CO** | 0 | 0 |
| **OT** | 1.1404 | 0 |
| **QM** | -0.1247 | 0 |
| **QS** | 0 | 0 |
| **QA** | 0 | 0 |
| **QP** | 0 | 0 |
| **QV** | 0 | 0 |
| **T1** | 0 | 0 |
| **T2** | 0 | 0 |
| **T3** | 0 | 0 |
| **T4** | 0 | 0 |
| **T5** | 0 | 0 |
| **T6** | 0 | 0 |
| **T7** | 0 | 0 |
| **T8** | 0 | 0 |
| **PR** | 0 | 1.4380 |
| **SO** | 1.4380 | 0 |
|  |  |  |

*For the legend of the node abbreviations, see Main Table 2.*


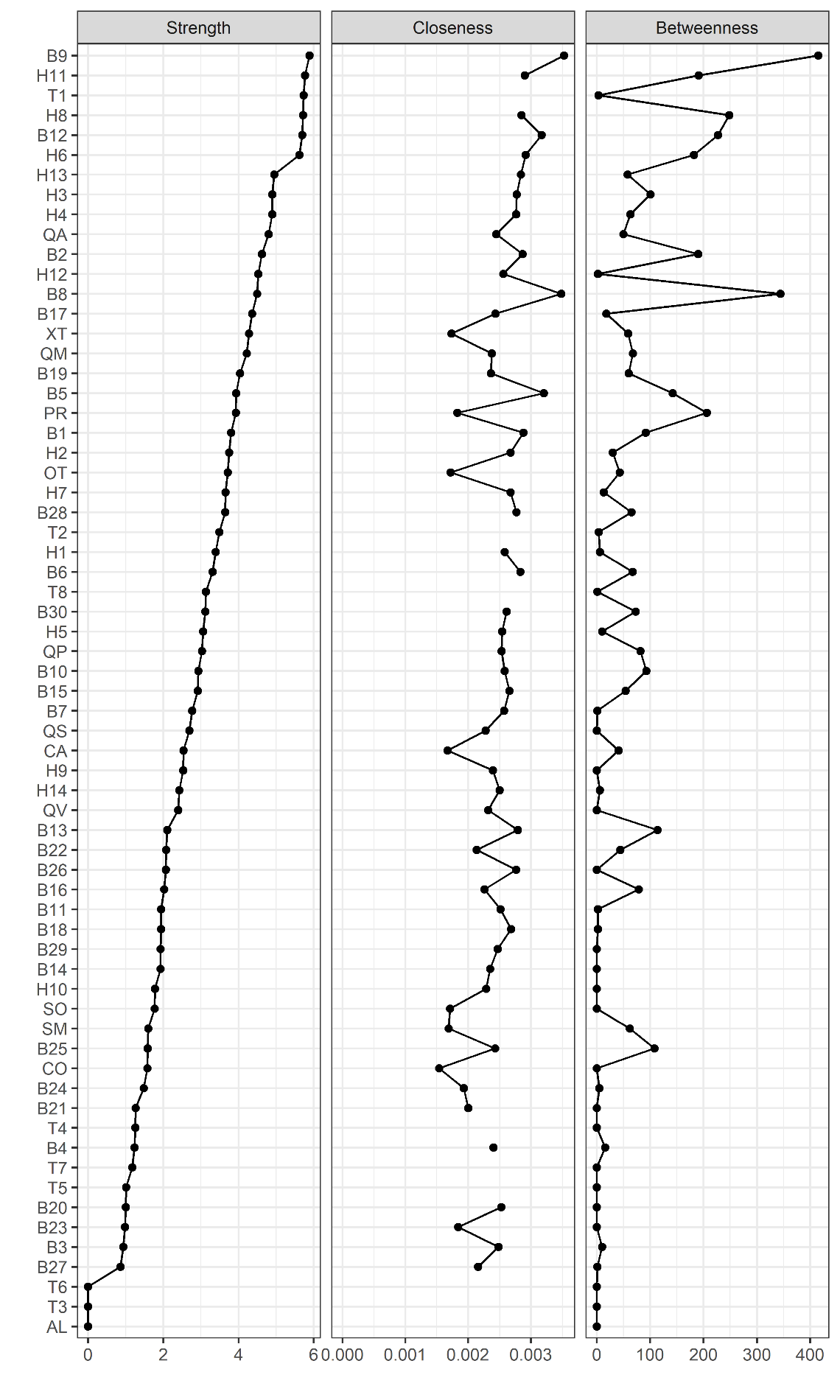


**Supplementary Figure 1.** Network model centrality indices. The network nodes are ordered by centrality index strength. For the legend of the node abbreviations, see Table 2.

**Supplementary Table 4.** Network model **s**trength centrality indices. The network nodes are ordered by centrality index strength. For the legend of the node abbreviations, see Table 2.

|  | **Strength** | **Closeness** | **Betweenness** |
| --- | --- | --- | --- |
| B9 | 5,89 | 0,0035 | 415 |
| H11 | 5,77 | 0,0029 | 191 |
| T1 | 5,73 |  | 3 |
| H8 | 5,72 | 0,0027 | 248 |
| B12 | 5,69 | 0,0031 | 227 |
| H6 | 5,62 | 0,0029 | 182 |
| H13 | 4,95 | 0,0028 | 58 |
| H3 | 4,90 | 0,0028 | 101 |
| H4 | 4,90 | 0,0027 | 63 |
| QA | 4,80 | 0,0024 | 50 |
| B2 | 4,62 | 0,0028 | 190 |
| H12 | 4,52 | 0,0025 | 2 |
| B8 | 4,50 | 0,0034 | 344 |
| B17 | 4,36 | 0,0023 | 18 |
| XT | 4,28 | 0,0017 | 59 |
| QM | 4,22 | 0,0023 | 68 |
| B19 | 4,04 | 0,0023 | 60 |
| B5 | 3,93 | 0,0032 | 142 |
| PR | 3,93 | 0,0018 | 206 |
| B1 | 3,81 | 0,0028 | 92 |
| H2 | 3,75 | 0,0026 | 30 |
| OT | 3,71 | 0,0017 | 43 |
| H7 | 3,66 | 0,0026 | 13 |
| B28 | 3,65 | 0,0027 | 65 |
| T2 | 3,49 |  | 4 |
| H1 | 3,39 | 0,0025 | 6 |
| B6 | 3,31 | 0,0026 | 67 |
| T8 | 3,13 |  | 1 |
| B30 | 3,12 | 0,0026 | 73 |
| H5 | 3,06 | 0,0025 | 10 |
| QP | 3,03 | 0,0025 | 82 |
| B10 | 2,93 | 0,0025 | 93 |
| B15 | 2,92 | 0,0026 | 54 |
| B7 | 2,77 | 0,0025 | 1 |
| QS | 2,70 | 0,0022 | 0 |
| CA | 2,54 | 0,0017 | 41 |
| H9 | 2,53 | 0,0023 | 0 |
| H14 | 2,43 | 0,0024 | 6 |
| QV | 2,39 | 0,0023 | 0 |
| B13 | 2,10 | 0,0028 | 114 |
| B22 | 2,07 | 0,0021 | 44 |
| B26 | 2,07 | 0,0027 | 0 |
| B16 | 2,02 | 0,0022 | 79 |
| B11 | 1,94 | 0,0025 | 2 |
| B18 | 1,94 | 0,0026 | 2 |
| B29 | 1,93 | 0,0024 | 0 |
| B14 | 1,92 | 0,0023 | 0 |
| H10 | 1,78 | 0,0023 | 0 |
| SO | 1,77 | 0,0017 | 0 |
| SM | 1,60 | 0,0017 | 62 |
| B25 | 1,58 | 0,0024 | 108 |
| CO | 1,58 | 0,0015 | 0 |
| B24 | 1,48 | 0,0019 | 5 |
| B21 | 1,26 | 0,0020 | 0 |
| T4 | 1,26 |  | 0 |
| B4 | 1,23 | 0,0024 | 16 |
| T7 | 1,17 |  | 0 |
| T5 | 1,01 |  | 0 |
| B20 | 1,01 | 0,0025 | 0 |
| B23 | 0,98 | 0,0018 | 0 |
| B3 | 0,93 | 0,0024 | 10 |
| B27 | 0,86 | 0,0021 | 1 |
| AL | 0,00 |  | 0 |
| T3 | 0,00 |  | 0 |
| T6 | 0,00 |  | 0 |
|  |  |  |  |


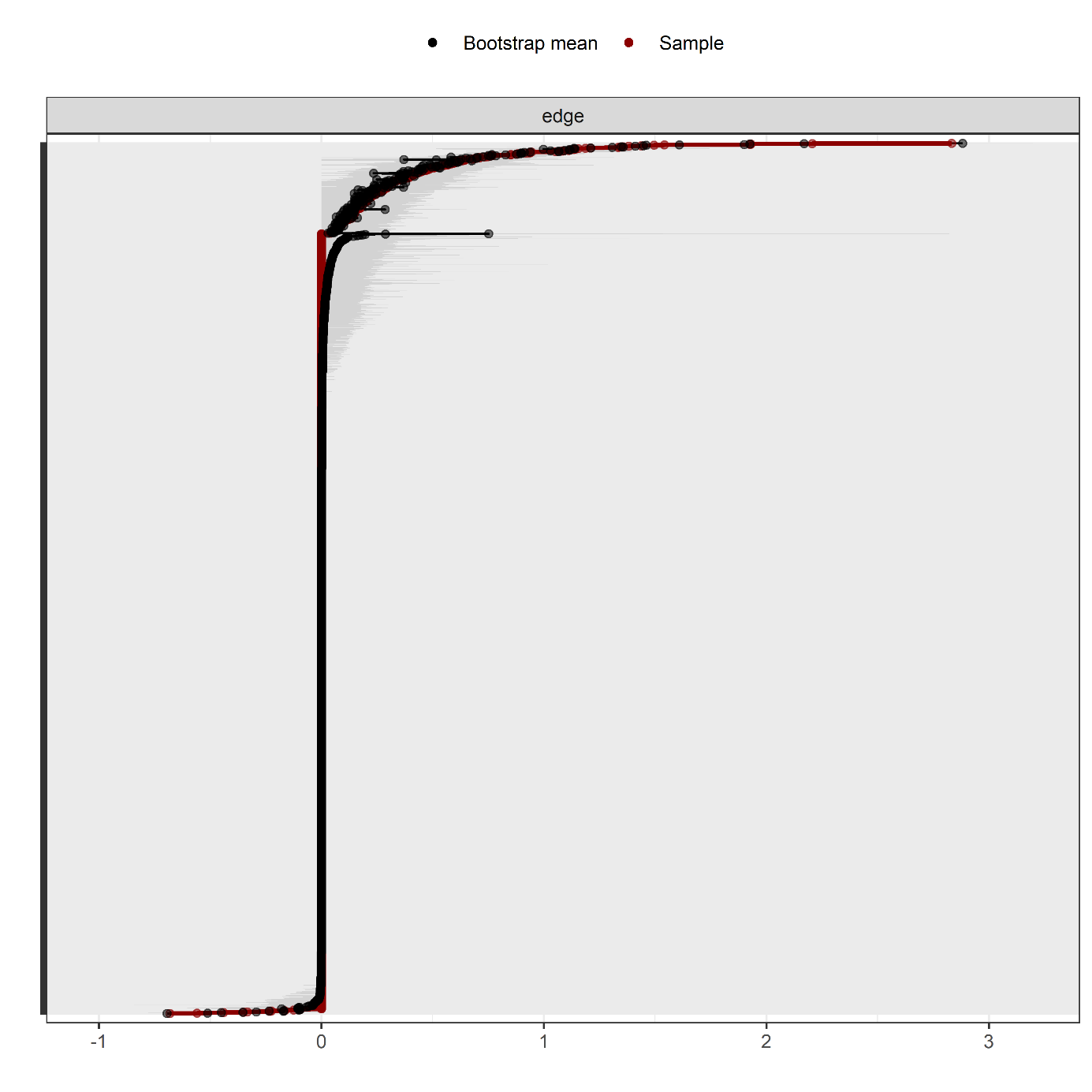


**Supplementary Figure 2.** Accuracy of network edge weights estimated with bootstrapped 95% confidence intervals.


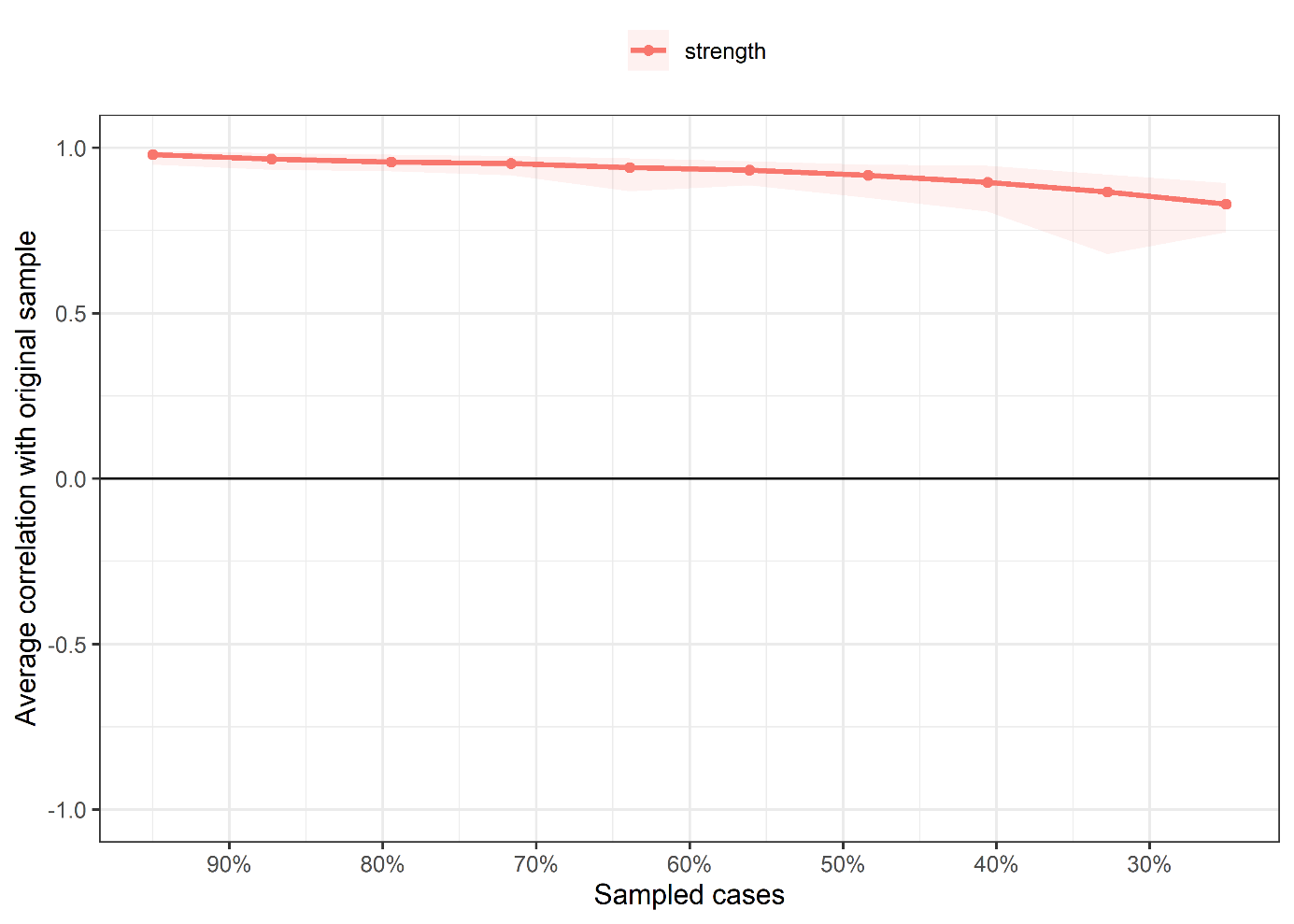


**Supplementary Figure 3.** Stability of the network strength centrality indices, calculated by case dropping subset bootstrap. The x-axis depicts the percentage of cases of the original sample used at each step. The y-axis depicts the average of correlations between the strength in the original network and the strength from the re-estimated networks after excluding increasing percentages of cases.


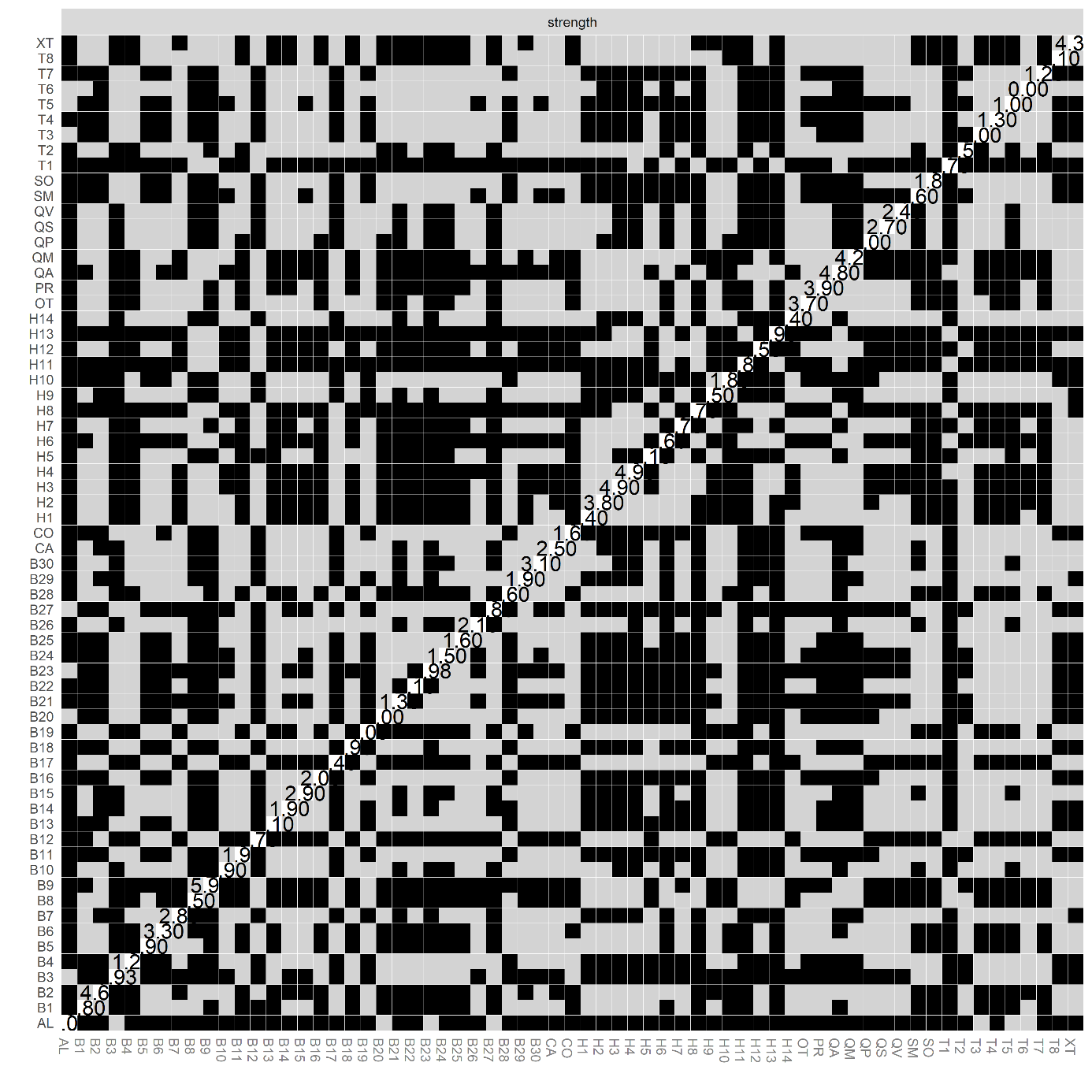


**Supplementary Figure 5.** Significant differences (p < 0.05) in network node strengths. For the legend of the node abbreviations, see Table 2.
